# A four-component modified Biginelli reaction: A novel approach for C-2 functionalized dihydropyrimidines

**DOI:** 10.3906/kim-2105-59

**Published:** 2021-09-21

**Authors:** Harsha NARKHEDE, Avinash DHAKE, Vaidhyanathan BALASUBRAMANIYAN

**Affiliations:** Department of Pharmaceutical Chemistry, S.M.B.T. College of Pharmacy, Dist-Nashik, India

**Keywords:** Biginelli reaction, green synthesis, 4-MCR, 2-Methylthio-1, 4-DHPMs, N-nucleophiles

## Abstract

A novel four component modified Biginelli reaction for the synthesis of C-2 functionalized dihydropyrimidines has been established. The approach uses assembly of less explored acetyl acetone with aromatic aldehyde, thiourea, and dimethyl sulphate to construct a novel 5-acetyl 2-methylthio dihydropyrimidine system, which works as an efficient well-designed intermediate for generating C-2 modified Biginelli libraries with nitrogen nucleophiles. Phenyl hydrazine, semicarbazide, and aryl semicarbazides are successfully used as N-nucleophiles to generate C-2 functionalized dihydropyrimidine derivatives, which fulfil the demands of active pharmacophore. Time economy, step economy, and a single pot reaction with moderate to excellent yield are the major advantages of this novel method.

## 1. Introduction

**Figure 1:**
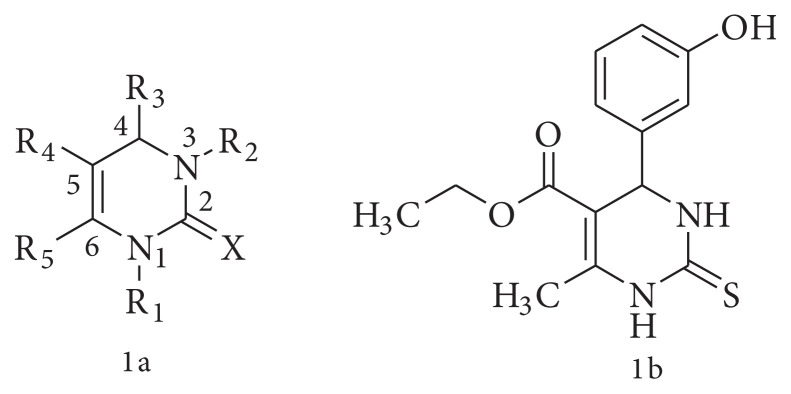
**a**-General structure of Biginelli adduct, **b**: Monastrol structure.

The synthesis of structurally diverse compounds has gained prime importance to generate molecular libraries in the drug discovery process. The focused issues for the novel developed reaction are the generation of these molecular libraries with excluding the drawbacks of classical reactions such as step-by-step process, tedious workup, use of toxic/expensive reagents/solvents/catalysts, long cyclisation period, and poor yield. Multicomponent reactions (MCR) are the hottest area in organic synthesis to generate scaffolds of drug like candidates [1,2]. Many pharmacophores have been generated from MCRs in medicinal chemistry, including praziquantel, nifedipine, and clopidogrel, to name a few [3,4]. Biginelli reaction, as a 3-CR was conceptualized in 1893 [5], and it gives simplicity of the reaction to generate dihydropyrimidines (DHPMs) with varied pharmacophores. From common organic reagents as well as the structural complexity with all six positions of pyrimidine nucleus, (Figure 1a) amenable to multiple chemical decorations, yielding a large library of compounds. The current surge of interest in this area in the last two decades is largely due to the diligent work of Kappe and his group [6,7]. Dihydropyrimidines (DHPMs) are the N-based heterocycle obtained from Biginelli MCR and have shown remarkable pharmacological activities [8–10]. The discovery of monastrol (Figure 1b) with its pharmacological activities in 1999 was a watershed moment [11,12]. Another fillip to Biginelli reaction (and other MCRs) was provided by the advent of green chemistry as a defining protocol for organic synthesis. The major attribute of MCR include, atom economy, step economy as well as saving energy and time resulting from simple work-up procedures [13–15].

A survey of recent literature revealed that modifications at the N1, C2, and N3 are particularly productive in offering drug-like candidates. Looking towards the libraries generated by the classical Biginelli reaction and the number of C2 modified DHPM derivatives showed tremendous scope for medicinal chemistry in the last decade [16]. In our earlier work on DHPMs [17,18], we noticed that working on DHPM areas deserve more effort. As revealed in the 2004 review of Kappe and several subsequent reviews, there have been abundant activities in DHPM-2-oxo/2-thio/2-amino with C5-ester. Unarguably, the activity spectrum of these 5-ester substituted DHPM – Monastrol led to the flood of publications on them, both DHPM-2-one and 2-thione. We also noticed that a handful of papers have been reported on 5-acetyl DHPMs. It appears to enrich the 5-acetyl DHPM chemistry, since they too display useful activities such as, anticancer, calcium channel blocker, antiviral, antiinflammatory, antitubercular, antioxidant, and antibacterial activities [9,10,19–21]. We also noticed that 5-acetyl DHPMs are conspicuously absent in many review articles and are less explored.

The 2-methylthio function has potential for a variety of nucleophilic displacements leading to biologically useful drug candidates. In this regard, current report highlights utility of this substrate by using few selected N-nucleophiles, viz. hydrazine hydrate, phenyl hydrazine, aryl semicarbazides, which will function as a lead for designing of new drug targets [16,22,23].

## 2. Materials and methods

### 2.1. General

All chemicals and reagents (AR) were procured from commercial sources. Melting points were determined by calibrated digital melting point apparatus (Make-Labline). TLC analysis was carried out on precoated Silica gel 60 F254 aluminum plates procured from Merck, Germany, and spots were visualized by UV light and/or by iodine vapors. Fourier-transform infrared (FT-IR) spectra for all the synthesized intermediates and final compounds were recorded on a JASCO 4100 FTIR spectrophotometer in the range of 4000–500cm^−1^. Proton nuclear magnetic resonance (^1^H NMR) spectra and carbon nuclear magnetic resonance (^13^C NMR) spectra were scanned using Bruker Avance Neo 500MHz spectrometer using DMSO-d_6_/CDCl_3_ as solvent. Chemical shift (δ) values are reported in ppm with TMS as an internal standard. Mass spectra were recorded on a Synapt-XS using the TOF MS ES+ method.

### 2.2. Synthesis

General procedure for synthesis of **1-(6-Methyl-2-methylsulfanyl-4-phenyl-1,4-dihydro-pyrimidin-5-yl)-ethanone: 5a** The mixture of benzaldehyde (**1a**, 1.01 mL, 0.01 mol), acetyl acetone (**2**, 1.03 mL, 0.01 mol) and thiourea (**3**, 0.91 g, 0.012 mol) was transferred to a reaction flask, and the flask was cooled to 0–5 °C in an ice bath. Dimethyl sulphate (4, 1.5 mL, 0.012 mol) was added drop wise over 10 min. The temperature rose to 60–65°C as a result of the ferocious reaction. Once the vigorous reaction ceased, 10 mL of ethanol was added and the reaction mixture was refluxed for 3–4 hours, along with stirring. The progress of reaction was monitored by TLC. The reaction mixture was cooled to 0°C and triturated with crushed ice and cold water. The solid separated was filtered off and washed with cold ethanol followed by ether. The crude product obtained was crystallized from hot ethanol to obtain compound **5a**.

Similarly, compounds **5b-e** were obtained by using aryl aldehydes viz. 3-hydroxy-4-methoxybenzaldehyde, 4-nitrobenzaldehyde, 4-chlorobenzaldehyde, and 2-hydroxy benzaldehyde respectively with acetyl acetone, thiourea and dimethyl sulphate by following similar reaction protocols.

**1-(6-Methyl-2-methylsulfanyl-4-phenyl-1,4-dihydro-pyrimidin-5-yl)-ethanone**, **(5a)** Yield: 78%, Ochre yellow solid; mp-128–129 °C; Rf-0.52 (Benzene: Ethyl acetate, 7:3). **FTIR (KBr, Ʋ_max_/cm^−1^):** 3278 (sec. NH), 3194 (Ar. CH), 3027 (Ali. CH), 1699 (C=O), 1616 (C=N), 1455 (Ar C=C), 1327 (C-N). **^1^H-NMR: **(500MHz, DMSO): δ 2.15 (s, 3H, C_6_-CH_3_), δ 2.24 (s, 3H-SCH_3_), δ 2.35 (s, 3H, CO-CH_3_) δ5.29–5.30 (s, 1H, C_4_-H), δ 7.09–7.46 (m, Ar.-CH, 5H), δ10.28 (s, 1H, -NH). **^13^C-NMR: **(500 MHz, DMSO): δ 194.67 (C=O), 173 (C=N), 126–139 (Ar. C=C), 110.36 (C=C), 53.68 (C_4_), 52.77 (S-CH_3_), 30.31 (CO-CH_3_), 18.15 (C_6_-CH_3_). ESI-MS: m/z calcd. for C_14_H_16_N_2_OS 260.4 found 264.4 [M+4]^+^

**1-[4-(4-hydroxy-3-methoxyphenyl)-6-methyl-2-(methylsulfanyl)-1,4-dihydropyrimidin-5-yl]ethenone, (5b)** Yield: 70%, Brown solid, mp-171–172 °C; Rf-0.24 (Benzene: Ethyl acetate, 7:3). **FTIR (KBr, Ʋ_max_/cm^−1^):** 3436 (-OH) 3290 (sec. NH), 3189, (Ar. CH), 3035 (Ali. -CH) 1711 (C=O), 1631 (C=N), 1512, 1454 (Ar C=C), 1326 (C-N), 1384 (C-O); **^1^H-NMR : **(500MHz, DMSO): δ 2.11 (s, 3H, C_6_-CH_3_), δ 2.28 (s, 3H-SCH_3_), δ 2.32 (s, 3H, CO-CH_3_), δ 5.20–5.21 (s, 1H, C_4_-H), δ 6.57–6.85 (m, Ar-CH, 3H), δ 9.03 (s, OH), 9.65 (s, 1H, -NH), δ10.20 (s, 1H, -NH) **^13^C-NMR : **(500 MHz, DMSO): δ 194.98 (C=O), 173.59 (C=N), 115–147 (Ar C=C), 110.02, 111.18 (C=C), 55.48 (C_4_), 53.62 (S-CH_3_), 30.03 (CO-CH_3_), 18.00 (C_6_-CH_3_)

**1-[6-methyl-2-(methylsulfanyl)-4-(4-nitrophenyl)-1,4-dihydropyrimidin-5-yl]ethenone**, **(5c)** Yield: 87%, Yellow grey solid, mp-192–193 °C; Rf-0.50 (Benzene: Ethyl acetate, 7:3); **FTIR (KBr, Ʋ_max_/cm^−1^):** 3270 (sec. NH), 3184 (Ar. -CH), 3078 (Ali. CH), 1699 (C=O), 1636 (C=N), 1464, 1418 (Ar C=C), 1347 (C-N), 1521 (C-NO_2_)

**1-[4-(4-Chloro-phenyl)-6-methyl-2-methylsulfanyl-1,4-dihydro-pyrimidin-5-yl]-ethanone**, **(5d)** Yield: 82%, buff solid; mp-177–178 °C; Rf-0.45 (Benzene: Ethyl acetate, 7:3) **FTIR (KBr, Ʋ_max_/cm^−1^):** 3279 (sec. NH), 3176 (Ar. CH), 3024 (Ali. CH), 1709 (C=O), 1619 (C=N),1490, 1455 (Ar. C=C), 1327 (C-N), 762 (C-Cl)

**1-[4-(3-hydroxyphenyl)-6-methyl-2-(methylsulfanyl)-1,4-dihydropyrimidin-5-yl]-ethenone, (5e)** Yield: 78%, Occur yellow solid, mp-176–177 °C; (Benzene: Ethyl acetate, 7:3) Rf-0.28 IR (KBr, Ʋmax/cm^−^^1^): 3450 (OH), 3296 (sec. NH), 3183, (Ar. CH), 3068 (Ali. -CH), 1719 (C=O), 1631 (C=N), 1509, 1445 (Ar. C=C), 1325 (C-N), 1380 (C-O)

**General procedure for synthesis of 1-(2-hydrazinyl-6-methyl-4-phenyl-1,4-dihydropyrimidin-5-yl) ethanone, (6a)**

To 10ml ethanolic solution of **5a** (260mg,1mmol), hydrazine hydrate (75mg, 1.5mmol) in 10ml ethanol was added drop wise and mixture was stirred under reflux conditions. The completion of reaction was monitored by TLC (2–4hrs). After the completion of reaction, crude mass obtained was cooled to room temperature and poured on to crushed ice. Obtained product was filtered off, washed with ether, dried, and recrystallized using ethanol to give product **6a**.

Similarly, compounds **6b-e** were obtained by using phenyl hydrazine/semicarbazide/aryl thiosemicarbazides.

**1-(2-hydrazinyl-6-methyl-4-phenyl-1,4-dihydropyrimidin-5-yl) ethenone, (6a)** Yield: 81%, Buff solid, mp-152–153 °C; Rf = 0.56 (Benzene: Ethyl acetate, 7:3); **FTIR (KBr, Ʋ_max_/cm^−1^):** 3432 (pri. NH_2_), 3283 (sec. NH), 3192 (Ar. CH), 3025 (Ali. CH), 1701 (C=O), 1607 (C=N), 1454 (Ar C=C), 1330 (C-N), 1184, 1115 (N-N). **^1^H-NMR: **(500MHz, DMSO-d_6_): δ 2.15 (s, 3H, C_6_-CH_3_), δ 2.34 (s, 3H, CO-CH_3_) δ5.31 (s, 1H, C_4_-H), δ 7.23–7.36 (m, Ar-CH, 5H), δ 9.78 (s, 1H, -NH), δ10.29 (s, 1H, -NH), δ 12.11 (s, 2H, -NH). **^13^C-NMR: **(500MHz, DMSO-d_6_): δ 194.66 (C=O), 173.98 (C=N), 126–144 (Ar C=C), 110.36 (C=C), 53.68 (C_4_), 30.31 (CO-CH_3_), 18.15 (C_6_-CH_3_). **ESI-MS:** m/z calcd. for C_13_H_16_N_4_O; 244.29 found 246 [M+2]^+^

**1-[6-methyl-4-phenyl-2-(2-phenylhydrazinyl)-1,4-dihydropyrimidin-5-yl]ethenone (6b)** Yield: 51%, Buff solid; mp-117–118 °C; Rf = 0.6 (Benzene: Ethyl acetate, 7:3); **FTIR (KBr, Ʋ_max_/cm^−1^):** 3356 (sec. NH), 3196, 3100 (Ar. CH), 3026 (Ali. CH)1712 (C=O), 1634 (C=N), 1600, 1558, 1495, 1455 (Ar C=C), 1339 (C-N), 1190, 1143, 1110 (N-N). **^1^H-NMR**: (500 MHz, DMSO-d_6_): δ 1.97 (s, 3H, C_6_-CH_3_), δ 2.07 (s, 3H, CO-CH_3_), δ5.30 (s, 1H, C_4_-H), δ 6.65–7.51 (m, Ar-CH, 10H), δ 8.93 (s, 1H, -NH), δ9.18 (s, 1H, -NH), δ 9.77 (s, 1H, -NH). **^13^C-NMR: **(500 MHz, DMSO-d_6_): δ 194.23 (C=O), 173.11 (C=N), 124–142 (Ar C=C), 112.40, 111.15(C=C), 55.87 (C_4_), 16.97 (CO-CH_3_), 16.27 (C_6_-CH_3_). ESI-MS: m/z calcd. for C_19_H_20_N_4_O; 320 found 324 [M+4]^+^

**2-(5-acetyl-6-methyl-4-phenyl-1,4-dihydropyrimidin-2-yl)hydrazinecarboxamide (6c)** Yield: 88%, Light brown solid; mp-190–192 °C; Rf-0.33 (Benzene: Ethyl acetate, 7:3); **FTIR (KBr, Ʋ_max_/cm^−1^):** 3400 (pri. NH2), 3217 (sec. NH), 3083 (Ar. CH), 1715 (keto C=O), 1681 (Amide C=O), 1632 (C=N), 1602, 1575, 1492 (Ar C=C), 1383 (C-N), 1182, 1113 (N-N) **^1^H-NMR**: (500 MHz, DMSO-d6): δ 2.11 (s, 3H, C6-CH3), δ 2.31 (s, 3H, CO-CH3) δ5.28–5.29 (s, 1H, C4-H), δ 6.78–7.72 (m, Ar-CH, 9H), δ 9.21 (s,1H,-NH), δ10.02 (s,1H,-NH), δ 9.81(s,1H, -NH), δ7.97(s,1H, -NH).**^13^C-NMR: **(500 MHz, DMSO-d6): δ 194.20 (C=O), δ 167.46 (C=N), δ 126.48–144.21 (Ar C=C), δ 109.71 (C=C), δ 55.87 (C4), δ 31.48 (CO-CH3), δ 18.97 (C6-CH3) **ESI-MS**: m/z calcd. for C_14_H_17_N_5_O_2_ 287.31 found 287 [M^+^]

**1-[6-Methyl-4-(4-nitro-phenyl)-2-(N′-phenyl-hydrazino)-1,4-dihydro-pyrimidin-5-yl]-ethanone (6d)** Yield: 92%, Red brown solid; mp-121–122 °C; Rf= 0.56 (Benzene: Ethyl acetate; 7:3); **FTIR (KBr, Ʋ_max_/cm^−1^):** 3397 (sec. NH), 3108 (Ar. CH), 3077 (Ali. CH), 1708 (C=O), 1632 (C=N), 1599, 1559, 1455 (Ar C=C), 1345 (C-N), 1186, 1108 (N-N), 1520 (CNO_2_). **^1^H-NMR**: (500 MHz, DMSO-d_6_): δ 1.98 (s, 3H, C_6_-CH_3_), δ 2.07 (s, 3H, CO-CH_3_), δ5.45 (s, 1H, C_4_-H), δ 6.67–7.96 (m, Ar-CH, 9H), δ 9.03 (s, 1H, -NH), δ9.39 (s, 1H, -NH), δ 9.81 (s, 1H, -NH), δ 9.91 (s, 1H, -NH). **^13^C-NMR: **(500 MHz, DMSO-d_6_): δ 194.23 (C=O), 173.34 (C=N), 123.11–146.75 (Ar C=C), 112.50, 110.51(C=C), 55.34 (C_4_), 17.14 (CO-CH_3_), 11.83 (C_6_-CH_3_). **ESI-MS:** m/z calcd. for C_19_H_19_N_5_O_3_; 365.5 found 363.15 [M-2]^+^

**2-[5-acetyl-4-(4-chlorophenyl)-6-methyl-1,4-dihydropyrimidin-2-yl]-*N*-(4-chlorophenyl)-hydrazinecarboxamide (6e)** Yield: 89%, Yellow buff solid; mp-156–157°C; Rf= 0.5 (Benzene: Ethyl acetate; 7:3); **FTIR (KBr, Ʋ_max_/cm^−1^):** 3425, 3313 (pri. NH_2_), 3215 (sec. NH), 3000 (Ar. CH), 1689 (keto C=O), 1653 (Amide C=O), 1611 (C=N), 1587, 1550, 1491. 1455 (Ar C=C), 1357 (C-N), 1182, 1117 (N-N).**^1^H-NMR: **(500 MHz, DMSO-d_6_): δ 2.13 (s, 3H, C_6_-CH_3_), δ 2.19 (s, 3H, CO-CH_3_) δ 5.31 (s, 1H, C_4_-H), δ5.93 (s,1H -NH) δ 7.24–7.46 (m, Ar-CH, 9H), δ 8.69 (s,1H,-NH), δ 9.80 (s,1H,-NH), δ 10.36 (s,1H, -NH). **^13^C-NMR: **(500 MHz, DMSO-d_6_): δ 194.57 (C=O), δ 174.15 (C=N), 155.78 (CO-NH) δ 124.44–144.82 (Ar C=C), δ 110.26 (C=C), δ 52.99 (C_4_), δ 30.41 (CO-CH_3_), δ 18.23 (C_6_-CH_3_). **ESI-MS**: m/z calcd. for C_20_H_19_Cl_2_N_5_O_2_; 432.30 found 437.21 [M+4]^+^

## 3. Results and Discussion

In hitherto reported literature, these 5-acetyl 2-methylthio DHPM derivatives have been obtained by initial synthesis of the corresponding thione by classical Biginelli reaction, followed by its conversion to the S-methyl derivatives by using methyl iodide or dimethyl sulphate [3,16,22,24] (Scheme a). The Atwal approach uses 3-CR to generate S-methyl function at C2 position using S-methyl iso-thiourea with poor yield. Also, this S-methyl iso-thiourea needs to be synthesized from thiourea and dimethyl sulphate, which increases one step for synthesis [25]. (Scheme b). Another route involves use of the classical Biginelli reaction followed by addtion of POCl_3_ to generate 2-chloro group in DHPMs [26] which subsequently reacted with N/O/C-nucleophiles to generate scaffolds of DHPM compounds. While various routes were studied, we envisioned that greening the process may be attempted in more than one aspect and, thus, scripted to run the reaction using a novel 4-component approach.

We first carried out the reaction with aryl aldehyde, acetyl acetone and thiourea to get the 5-acetyl DHPM-2-thione and treated it with Me_2_SO_4_ rather than methyl iodide (as a greener choice), followed by the classical approach, i.e. Scheme a. It worked well. Further, the modification has been done by the Atwal approach using aryl aldehyde, acetyl acetone, and S-methyl iso-thiourea, i.e. cheme b as a 3-component reaction to obtain 5-acetyl-2-methylthio DHPMs, but this showed poor yield as compared to Scheme a.

To continue work on DHPMs, a simpler and more elegant a novel 4-MCR method was developed for synthesizing 5-acetyl-2-methyl thio DHPMs in a single pot, single step synthesis using aryl aldehyde, acetyl acetone, thiourea, and Me_2_SO_4_ in good to excellent yield. It too worked well with comparable yields. We then reasoned that designing a 4-component reaction could offer a much better protocol. The reaction of aryl aldehydes **1a–e**, acetylacetone (1,3-dicarbonyl compound) **2**, thiourea **3** and dimethyl sulphate **6** was attempted in a one pot reaction to get **5a–e** in a single step. (Table 1, Novel 4-MCR approach). After adding dimethyl sulphate, vigorous reaction was noted, and the temperature of reaction increased up to 60–65 °C. Once the vigorous reaction had ceased, ethanol was added to the reaction mixture, which was then stirred with reflux to complete the reaction.

To our delight, the paradigms gave an acceptable yield for 4-CR. The structure was fully confirmed with analysis/spectral data. Repeating the protocol with other aldehydes confirmed the reliability of the reactions. The general applicability of this 4-component reaction created our new series of 5-acetyl-2-methylthio DHPMs and further processed to replace S-methylthio group with N-nucleophiles viz. hydrazine hydrate, phenyl hydrazine, semicarbazide and aryl semicarbazide offers C-2 functionalized novel DHPM-nucleophile molecular hybrids. Among the synthesized series of 5-acetyl-2-methythio DHPMs, selected compounds **5a**, **5c**, and **5d** produced a series of newly designed target compounds **6a–e** after treatment with various nucleophiles (Table 2). We have successfully synthesized hybrids of C2 functionalized DHPMs with N-nucleophiles using the S-methylthio system as an intermediate compound. These compounds were also fully characterized. By using this approach, assorted C2 functionalized DHPM libraries can be generated using N/O nucleophiles as well. These experiments illustrate a greener approach to obtain C2 substituted-DHPM derivatives of versatile bioactivity. Work on these lines is in progress in our labs.

To propose an idea about the mechanism, several observations have been made which provide a clue to the probable pathway. The reaction involves post modification of the Biginelli DHPMs using acetyl acetone, thiourea, aromatic aldehyde and dimethyl sulphate as a one-pot 4CR. There are two plausible reaction mechanisms, the first one is, the initial formation of Biginelli thione, which subsequently reacts with dimethyl sulphate to offer 2-methylthio DHPMs. The second possibility includes initial reaction of S-methylation of thiourea to give S-methyl iso-thiourea which itself participates in the Biginelli reaction. By considering various observations, the reaction pathway can be proposed as follows-

**Figure 2:**
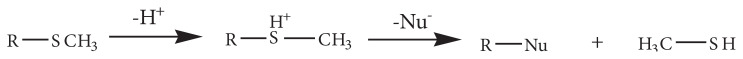
Proposed reaction mechanism.

When the Acetyl acetone, S-methyl iso-thiourea and aryl aldehyde are mixed, no exothermic reactions are observed. When aromatic aldehyde, ethyl acetoacetate and thiourea were mixed with ethanol, reaction did not proceed. When all four reactants, are mixed together, the exothermic reaction was noted, due to formation of S-methyl iso-thiourea, S-MITU. (Confirmed with TLC). Due to rise in temperature, reaction gets initiated. Exothermic reaction (60–65°C) was due to formation of S-MITU from thiourea and dimethyl sulphate. The reaction is quite exothermic and needs to be controlled [27]. Thus, an assumption can be obtained from the above observations, that the reaction follows the second route for the synthesis of 2-methylthio DHPMs i.e. the S-MITU formation is the first step followed by the Biginelli with the established protocol [28]. The next step is the step of the nucleophilic substitution reaction. After addition of N-nucleophiles, to 5-acetyl 2-methylthio DHPM derivatives, a peculiar odour of methyl mercaptan, CH_3_SH (odour of rotten cabbage) was reported [29]. This confirms the elimination of CH_3_SH. This signifies the probable mechanism, i.e. replacement of S-methyl function with nucleophile, Figure 2.

Improvements in % yield, time economy, step economy and mild reaction conditions, are the key features of the novel MCR. The reported classical approaches to generate C2 functionalised DHPMs involve 3–4 step synthesis to reach final products along with use of toxic reagents like POCl_3_ and methyl iodide. This novel 4-CR method is advantageous over the earlier reported methods in relation to number of steps, time, and avoiding use of nongreener agents such as POCl_3_. The method also uses dimethyl sulphate as a methylating agent, instead of the expensive, unstable compound methyl iodide, which most researchers used earlier [3,16,22,24,30]. Thus, newly designed MCR also avoids the use of these toxic/expensive reagents, which are some added benefits of this novel approach. The 2-methylthio system in DHPMs has proven its scope as a versatile intermediate for generation of drug like molecules by reaction with N/O-nucleophiles [9,16]. In this regard, we have reported use of 2-methylthio DHPMs reaction with N-nucleophiles which will be screened as an active pharmacological motif. A preliminary investigation is under way at NCI, USA for anticancer screening. In comparison, we used a greener and more economical reaction protocol for synthesis of C-2 functionalised DHPM libraries. All the compounds were screened for FT-IR analysis, and the presence of functional groups was confirmed. Prototype structures of compounds from 2-methylthio DHPMs series, viz. compounds **5a, 5b** and C-2 functionalised DHPMs **6a–e** were confirmed through ^1^H NMR, ^13^C NMR, and mass spectroscopy. The analysis of spectroscopic data confirms the structures of newly syntheiszed molecules. [Refer Supplementary information Figure: 1S–21S]

The synthesized substrate (C-2 functionalized DHPM i.e. 2-methylthio dihydropyrimidine) has diverse scope for generation of libraries. The synthesis of novel 5-acetyl 2-methylthio DHPM derivatives were reported for the first time via a novel four component modified Biginelli reaction. Modified novel reaction was also compared with earlier reported classical Biginelli and Atwal modified reaction. In comparison to earlier reported reaction protocols, novel method allows one pot, single step for generation of intermediate, i.e. 5-acetyl 2-methylthio dihydropyrimidine. Thus, novel method demonstrates more benefits over earlier reported reaction protocols.

## 4. Discussion

In continuation of our interest in DHPM derivatives, herein we report an efficient and novel method for the synthesis of 5-acetyl 2-methylthio DHPMs. The method allows more efficient, time-saving, one pot and single step reaction with moderate to excellent yield. The importance of 2-methylthio function in DHPMs was emphasized because it serves as a good leaving group and can react with N/O-nucleophiles. This led to the synthesis of C2-functionalsed DHPMs with use of selected N-nucleophiles, and the conjugates formed may fulfil the demand of active pharmacophores. Use of one pot 4-MCR increases efficiency of synthesis, and incorporation of N-nucleophiles introduces diverse complexity in the DHPM nucleus, which will be the potential future targets (drug discovery pipeline) for development of APIs.

## Supplementary Information

**Article Category:** Research paper

**Title: A four-component modified Biginelli reaction: A novel approach for C-2 functionalized dihydropyrimidines**

Author(s): Harsha I. Narkhede*, Avinash S Dhake, Balasubramaniyan V

Department of Pharmaceutical chemistry, S.M.B.T. College of Pharmacy, Dhamangaon, Nashik, M.S. India-422403

Prototype intermediate compounds and all final compounds are characterized by FT-IR, ^1^H-NMR, ^13^C NMR and mass spectrometry. Presence of absorption band in the range at 1696–1720 cm^−1^ reveals the presence of keto carbonyl in the structure. Ureide N-H band was located at 3290–3278 cm^−1^ and C=N is confirmed by presence of absorption band at 1607–1636 cm^−1^ in all **5a–e** and **6a–e**. Presence of N-N bond in **6a–e** is confirmed by presence of strong absorption band at 1105–1120 cm^−1^ whereas absorption band at 3400–3500 cm^−1^ confirms the presence of primary amine in compound **6a** and **6e**. Absorption band at 1653–1681 cm^−1^ shows presence of amide carbonyl in compounds **6c** and **6e**. In ^1^H NMR spectra, two singlet δ 1.97–2.15ppm, δ 2.20–2.35ppm designates the methyl proton at C-6 and acetyl proton at C-5 position respectively. Similarly, a singlet at δ 5.5.20–5.31 ppm is attributed to the C-4 proton of DHPM ring. Appearance of singlet due to S-Methyl at δ 2.25 ppm in all series **5** compounds, disappearance of S-methyl and appearance of extra -NH singlet in compounds **6a–e** represents displacement of S-methyl by N-nucleophiles. Presence of three -NH peaks at δ 9.78, 10.29, 12.11 ppm in **6a** represents two -NH proton and -NH_2_ proton peaks respectively. Also, singlet at δ 8.93, 9.18 and 9.77 ppm represent three -NH peaks in **6b** and **6d**. For compounds substituted with thiosemicarbazide as a nucleophile **6c** showed three -NH peaks at δ 7.97, 9.21, 9.81 ppm and -NH_2_ peak at δ10.02 ppm. In all compounds, a multiplet at δ 6.57–7.72 ppm reveals identity of aromatic protons. In ^13^C NMR, δ 18.5, 30.31, 55–58, 110, 125–150, 173 and 194 ppm show presence of C6-CH_3_, keto-CH_3_, C_4_-carbon, C=C, Ar. C=C, C=N and C=O respectively. Presence of S-methyl is confirmed by presence of δ 52–53 ppm in compound **5a** and **5b**. Absence S-methyl group in series of compounds **6a–e** confirms the displacement of S-methyl with N-nucleophiles. Molecular ion peaks, base peaks and further fragments of Mass analysis shows structural resemblance with molecular weight of compounds.

Figure 1S 1-(6-Methyl-2-methylsulfanyl-4-phenyl-1,4-dihydro-pyrimidin-5-yl)-ethanone, **(5a)**
**^1^H-NMR: **(novel MCR approach)

Figure 2S **^13^C-NMR:**1-(6-Methyl-2-methylsulfanyl-4-phenyl-1,4-dihydro-pyrimidin-5-yl)-ethanone **(5a)**

Figure 3S ESI-MS: 1-(6-Methyl-2-methylsulfanyl-4-phenyl-1,4-dihydro-pyrimidin-5-yl)-ethanone **(5a)**

Figure 4S **ESI-MS:** 1-(6-Methyl-2-methylsulfanyl-4-phenyl-1,4-dihydro-pyrimidin-5-yl)-ethanone **(5a)**

Figure 5S **^1^H-NMR:**1-[4-(4-hydroxy-3-methoxyphenyl)-6-methyl-2-(methylsulfanyl)-1,4-dihydropyrimidin -5-yl]ethenone, **(5b)**

Figure 6S ^13^C-NMR : (5b)

Figure 7S **^1^H-NMR:**1-(2-hydrazinyl-6-methyl-4-phenyl-1,4-dihydropyrimidin-5-yl) ethenone, **(6a)**

Figure 8S **^13^C-NMR:** 1-(2-hydrazinyl-6-methyl-4-phenyl-1,4-dihydropyrimidin-5-yl) ethenone, **(6a)**

Figure 9S ESI-MS: 1-(2-hydrazinyl-6-methyl-4-phenyl-1,4-dihydropyrimidin-5-yl) ethenone, **(6a)**

Figure 10S **^1^H-NMR**: 1-[6-methyl-4-phenyl-2-(2-phenylhydrazinyl)-1,4-dihydropyrimidin-5-yl]ethenone **(6b)**

Figure 11S **^13^C-NMR:** 1-[6-methyl-4-phenyl-2-(2-phenylhydrazinyl)-1,4-dihydropyrimidin-5-yl]ethenone **(6b)**

Figure 12S ESI-MS: 1-[6-methyl-4-phenyl-2-(2-phenylhydrazinyl)-1,4-dihydropyrimidin-5-yl]ethenone **(6b)**

Figure 13S 2-(5-acetyl-6-methyl-4-phenyl-1,4-dihydropyrimidin-2-yl)hydrazinecarboxamide (6c) ^1^H-NMR:

Figure 14S ^13^C-NMR: 2-(5-acetyl-6-methyl-4-phenyl-1,4-dihydropyrimidin-2-yl)hydrazinecarboxamide (6c)

Figure 15S ESI-MS: 2-(5-acetyl-6-methyl-4-phenyl-1,4-dihydropyrimidin-2-yl)hydrazinecarboxamide (6c)

Figure 16S **^1^H-NMR**: 1-[6-Methyl-4-(4-nitro-phenyl)-2-(N′-phenyl-hydrazino)-1,4-dihydro-pyrimidin-5-yl]-ethanone **(6d)**

Figure 17S ^13^C-NMR: (6d)

Figure 18S ESI-MS: (6d)

Figure 19S **^1^H-NMR:** 2-[5-acetyl-4-(4-chlorophenyl)-6-methyl-1,4-dihydropyrimidin-2-yl]-*N*-(4-chlorophenyl)-hydrazinecarboxamide (6e)

Figure 20S ^13^C-NMR: (6e)

Figure 21S ESI-MS: (6e)

## Figures and Tables

**Scheme 1:**
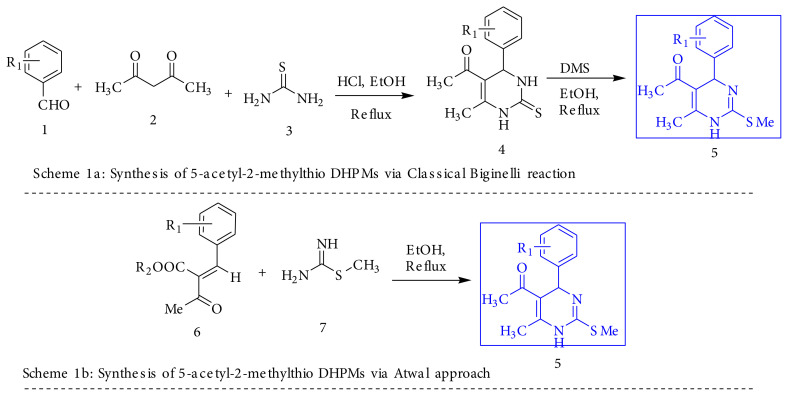
Synthesis of 5-acetyl-2-methylthio DHPMs via earlier approaches.

**Table 1:** Synthesis of 5-acetyl-2-methylthio DHPMs via novel 4-MCR approach.

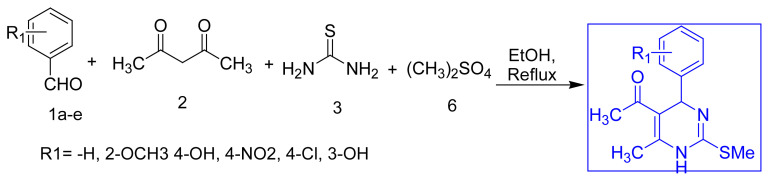						
Entry	Code	R_1_	Time (h)	Rf^a^	% Yield	mp °C
1	5a	H	5	0.52	78	128–129
2	5b	2-OH, 3-OCH_3_	4.5	0.24	70	171–172
3	5c	4-NO_2_	5	0.50	87	192–193
4	5d	4-Cl	5.5	0.45	82	177–178
5	5e	3-OH	5	0.28	78	176–177

a Solvent system: Benzene: Ethyl acetate (7:3); Visualizing agent: I^2^ vapors: yellow spots, / short UV 254 nm: purple spots

**Table 2:** Synthesis of C2 functionalised DHPMs with N-nucleophiles.

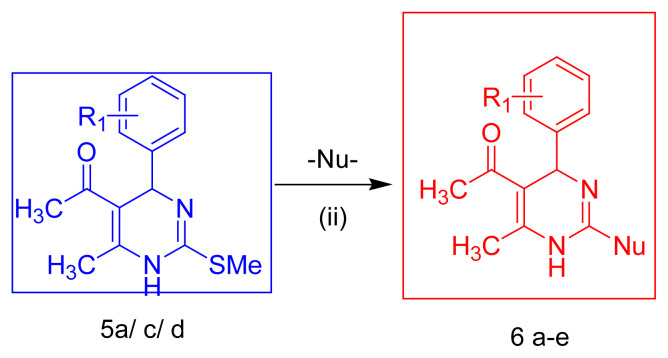							
Entry	Code	R_1_	Nu-	Time(h)	Rf^a^	%Yield	mp °C
6	6a	-H	-NHNH_2_	2.5	0.56	81	152–153
7	6b	-H	-NHNHPh	4	0.6	51	117–118
8	6c	-H	-NHNH C(O)NH_2_	4	0.33	88	191–192
9	6d	4-NO_2_	-NHNHPh	4.5	0.56	92	121–122
10	6e	4-Cl	NHNHC(O)NH (4-Cl)Ph	4	0.50	89	156–157

a Solvent system: Benzene: Ethyl acetate (7:3); Visualizing agent: I^2^ vapors: Yellow spots, / Short UV 254 nm: purple spots, Nu^−^= -NHNH_2_; -NHNHPh; -NHNHC(O)NH_2_ -NHNHC(O)NH-(4-Cl) Ph.
