# Supplementary figures and images for: A four-component modified Biginelli reaction: A novel approach for C-2 functionalized dihydropyrimidines
Source: Turk J Chem. 2021 Sep 21;45(6):1980–7. doi: 10.3906/kim-2105-59 (PMC10734754; doi:10.3906/kim-2105-59)

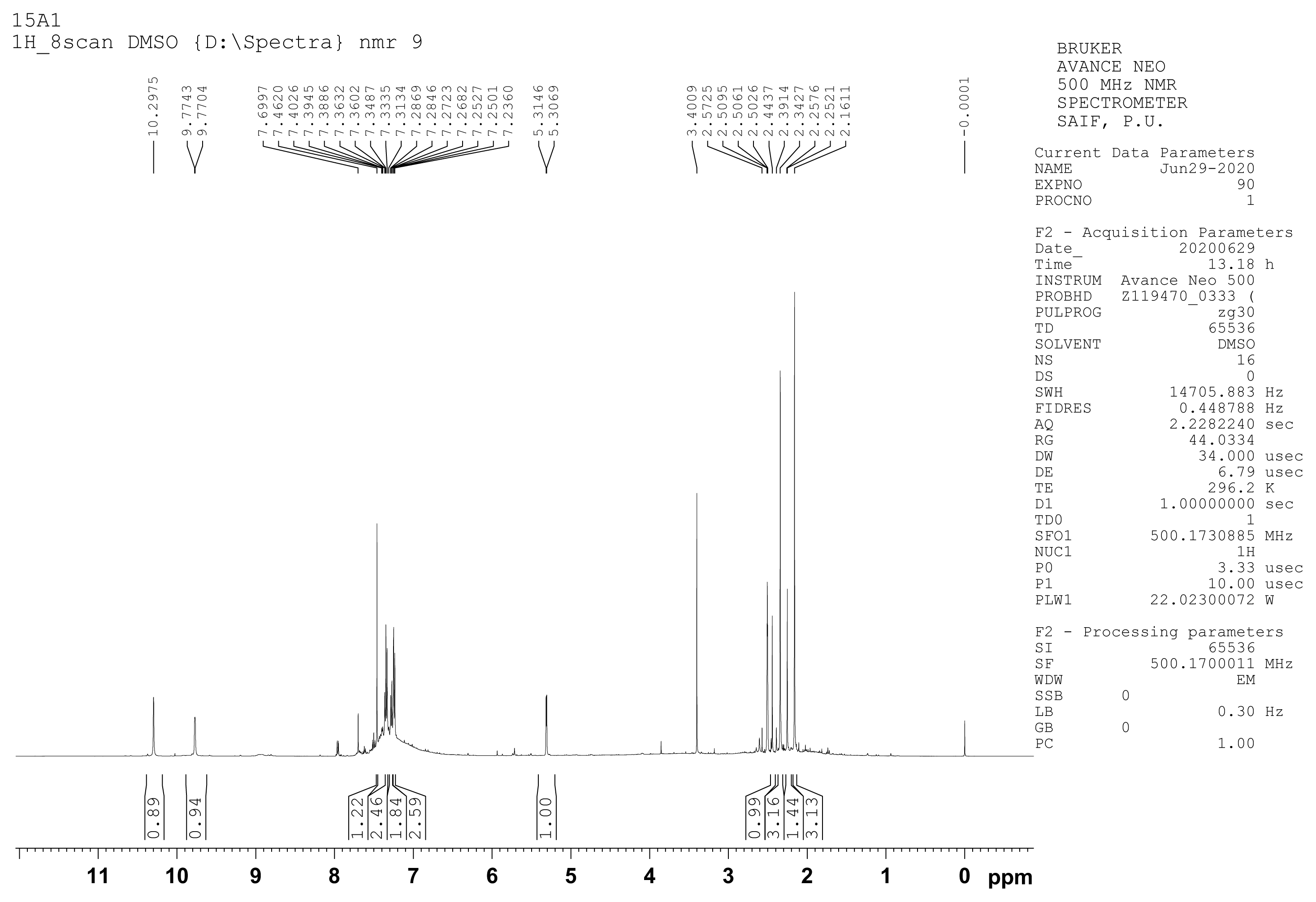

Supplement: Figure 1S — 1-(6-Methyl-2-methylsulfanyl-4-phenyl-1,4-dihydro-pyrimidin-5-yl)-ethanone, (5a) 1H-NMR: (novel MCR approach) [file turkjchem-45-6-1980s1.tif]

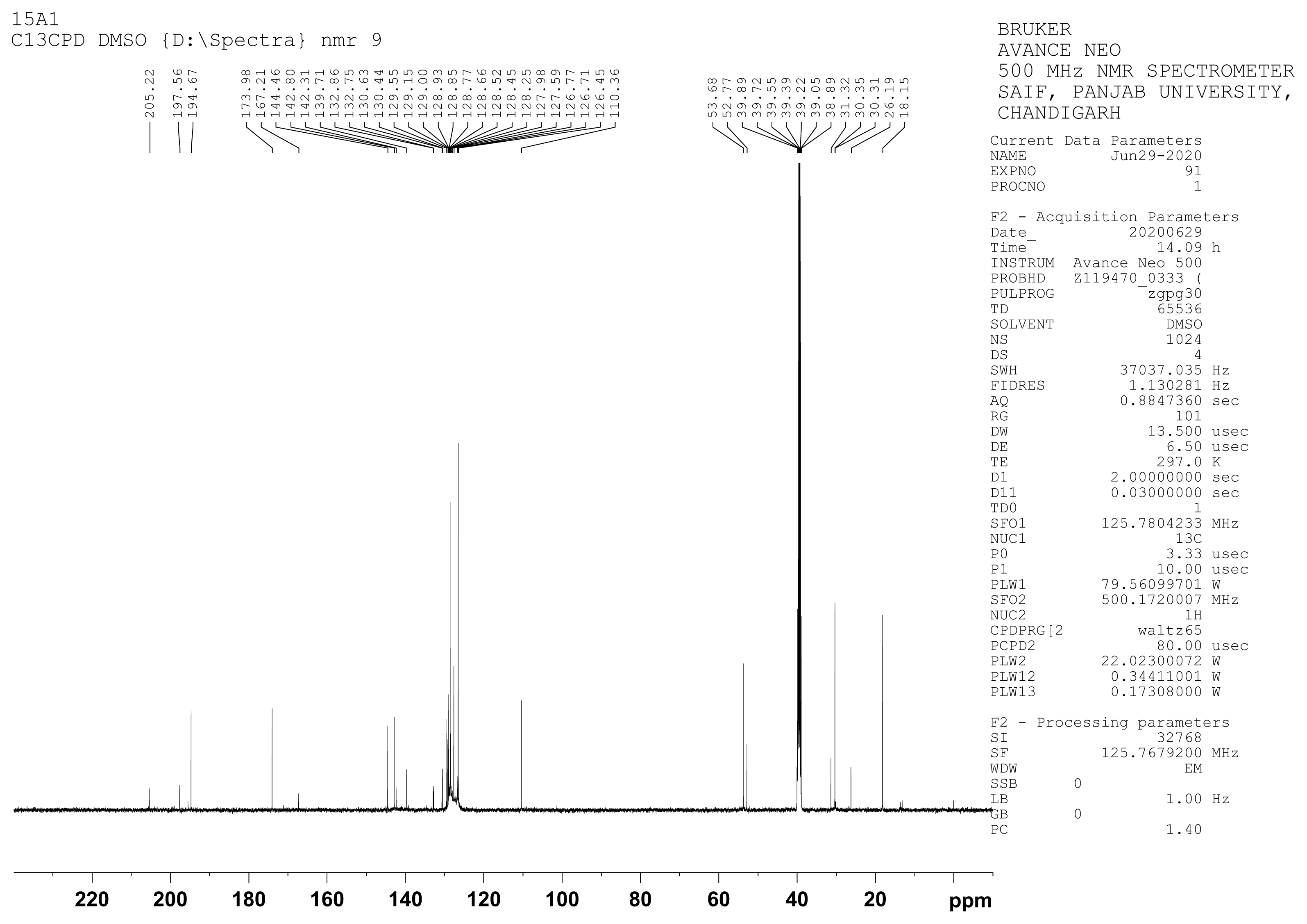

Supplement: Figure 2S — 13C-NMR:1-(6-Methyl-2-methylsulfanyl-4-phenyl-1,4-dihydro-pyrimidin-5-yl)-ethanone (5a) [file turkjchem-45-6-1980s2.tif]

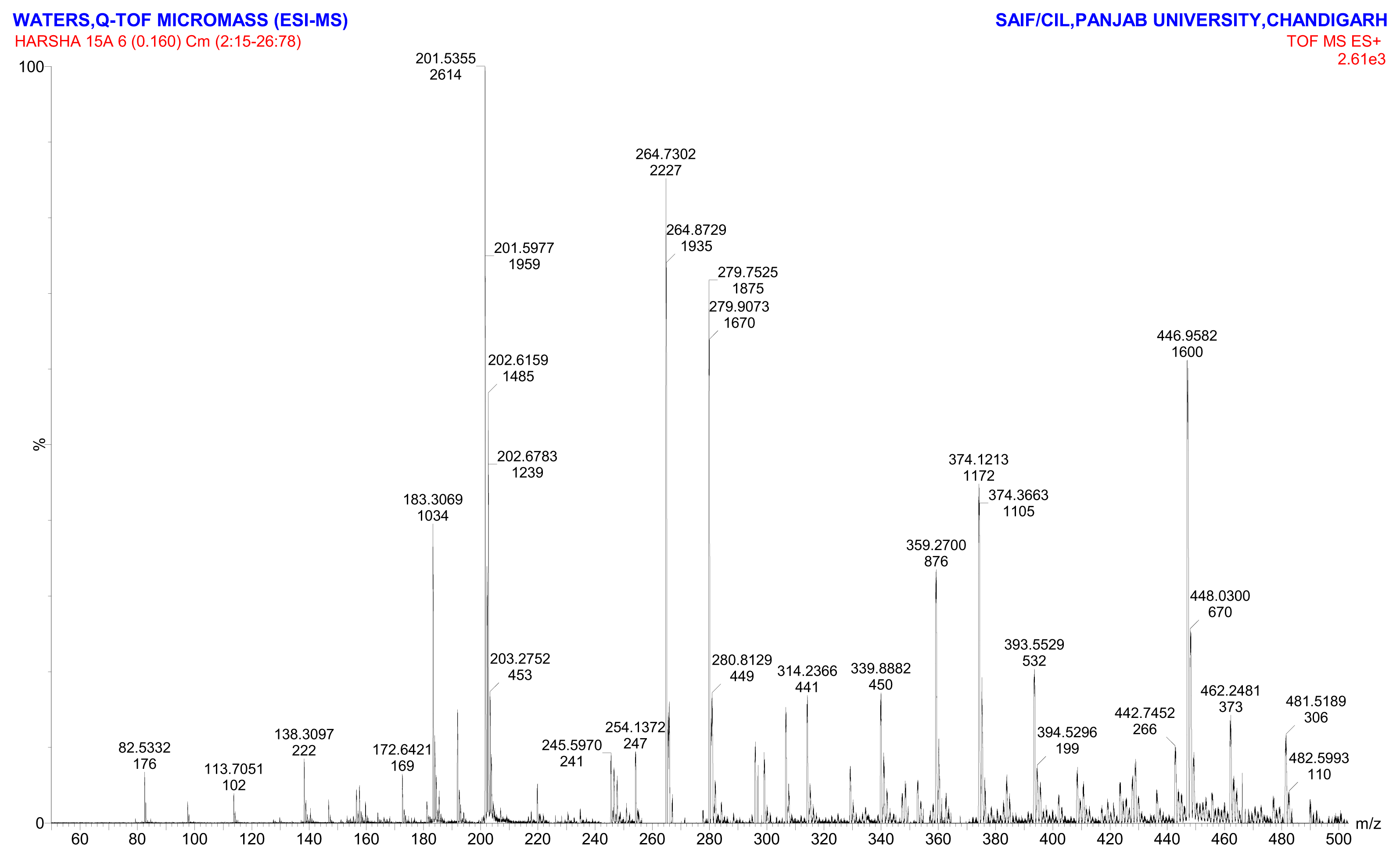

Supplement: Figure 3S — ESI-MS: 1-(6-Methyl-2-methylsulfanyl-4-phenyl-1,4-dihydro-pyrimidin-5-yl)-ethanone (5a) [file turkjchem-45-6-1980s3.tif]

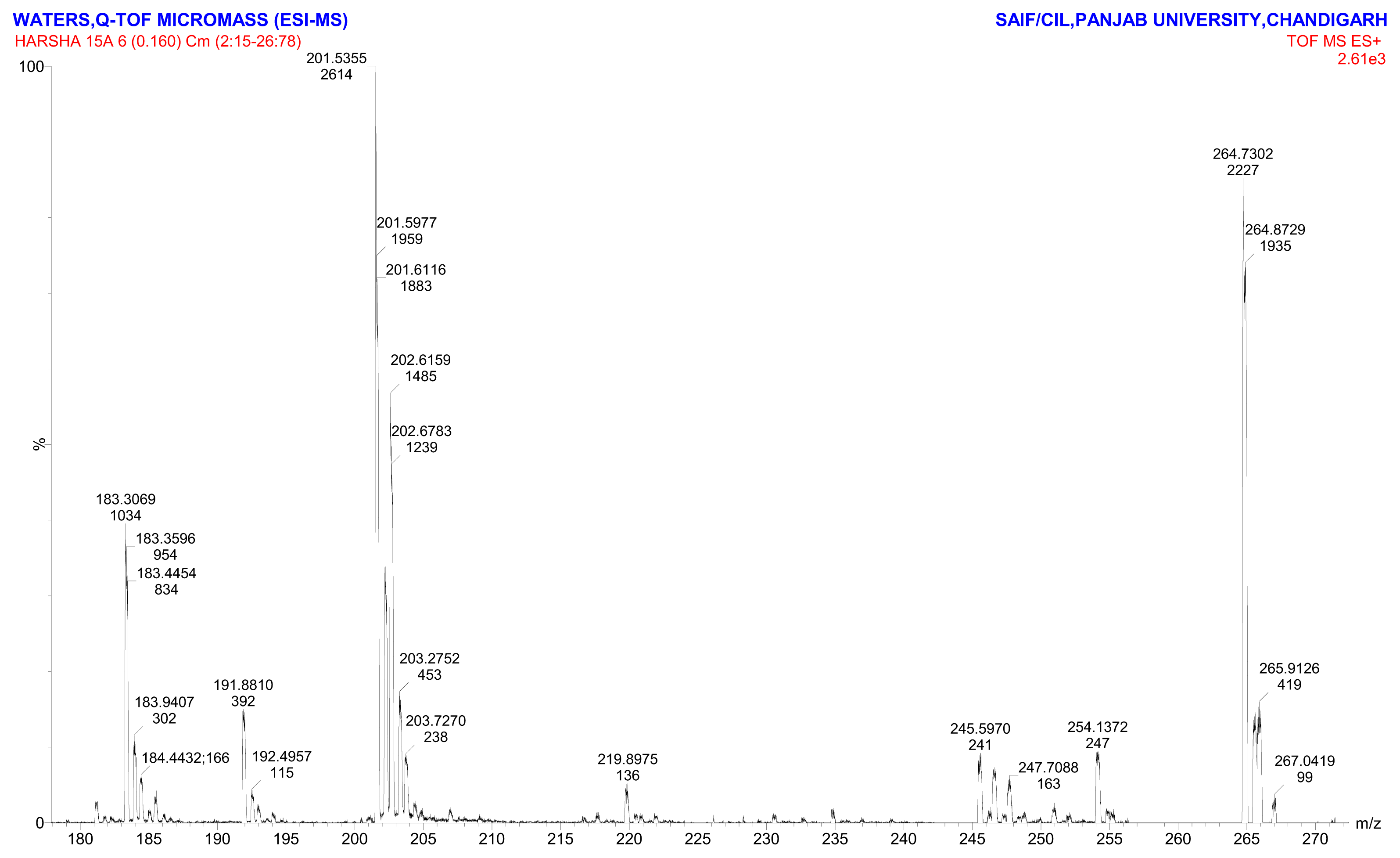

Supplement: Figure 4S — ESI-MS: 1-(6-Methyl-2-methylsulfanyl-4-phenyl-1,4-dihydro-pyrimidin-5-yl)-ethanone (5a) [file turkjchem-45-6-1980s4.tif]

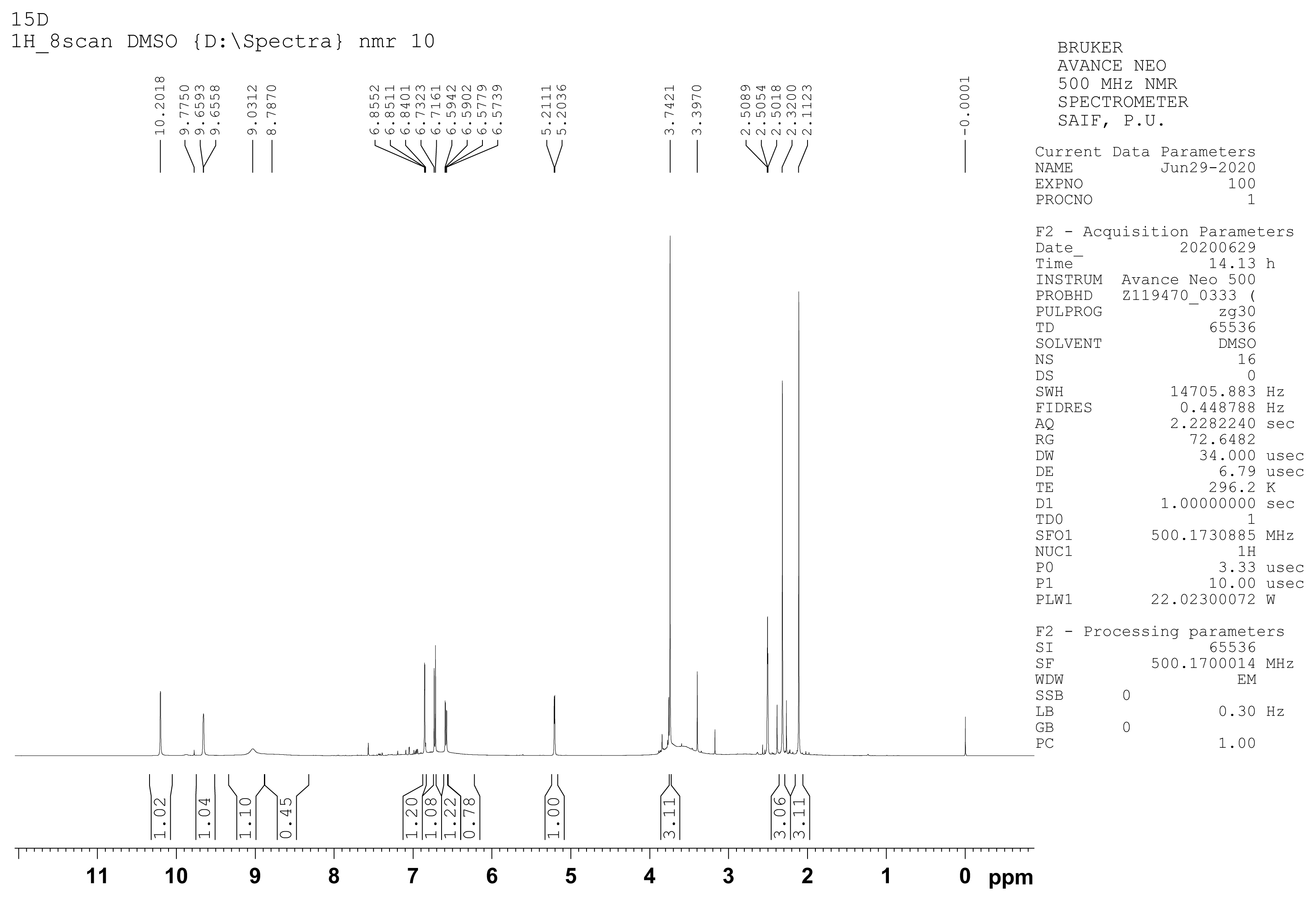

Supplement: Figure 5S — 1H-NMR:1-[4-(4-hydroxy-3-methoxyphenyl)-6-methyl-2-(methylsulfanyl)-1,4-dihydropyrimidin -5-yl]ethenone, (5b) [file turkjchem-45-6-1980s5.tif]

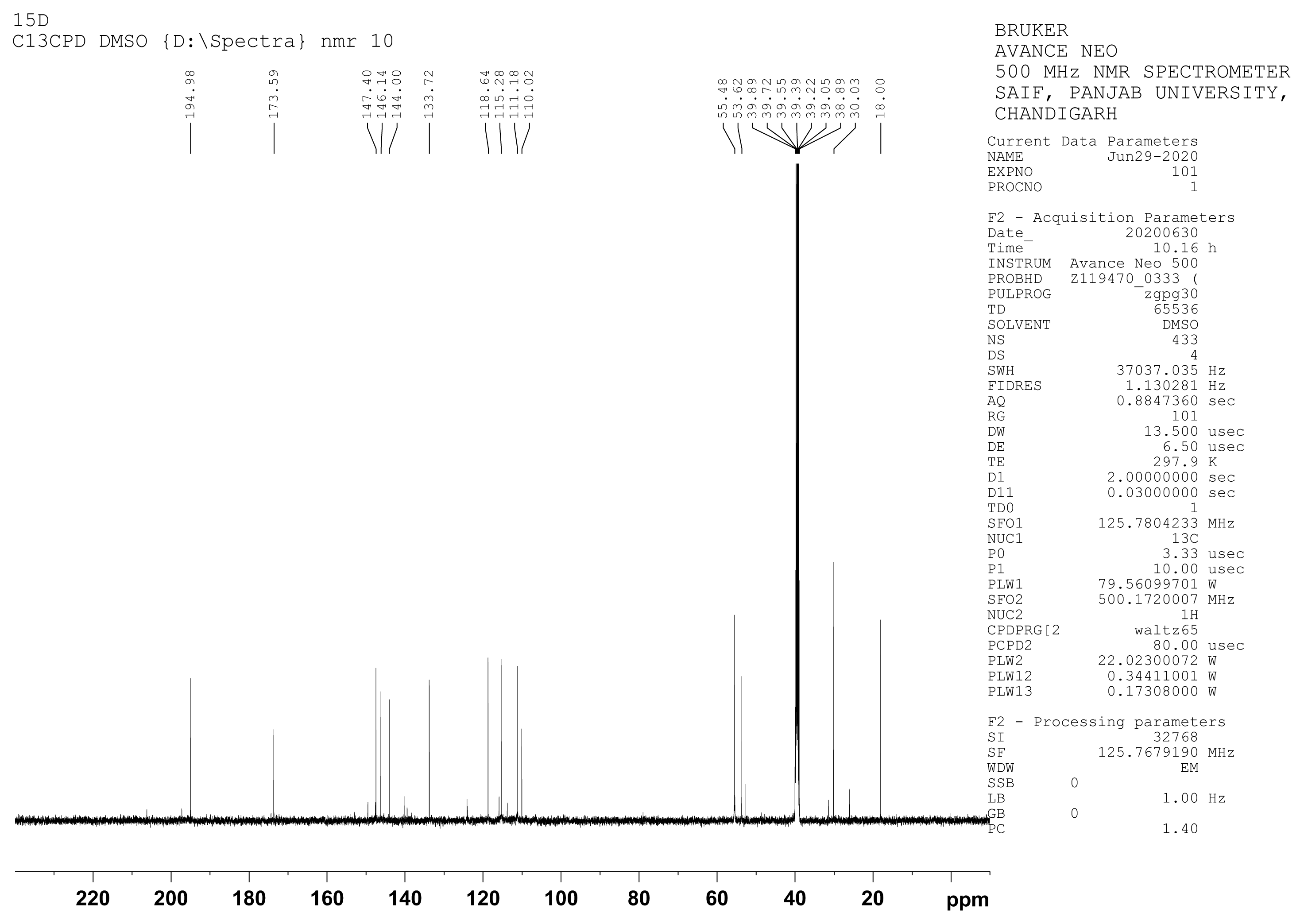

Supplement: Figure 6S — 13C-NMR : (5b) [file turkjchem-45-6-1980s6.tif]

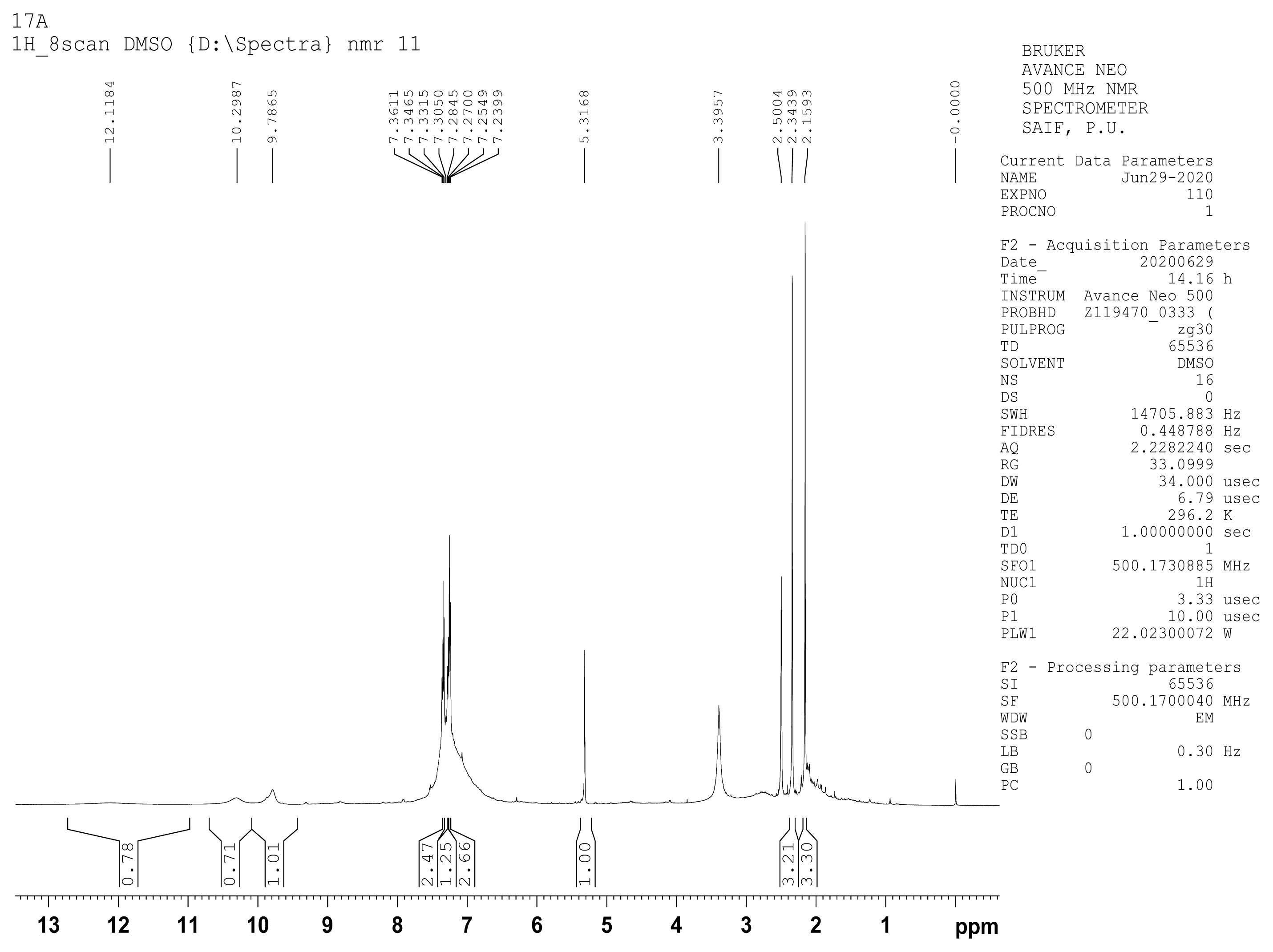

Supplement: Figure 7S — 1H-NMR:1-(2-hydrazinyl-6-methyl-4-phenyl-1,4-dihydropyrimidin-5-yl) ethenone, (6a) [file turkjchem-45-6-1980s7.tif]

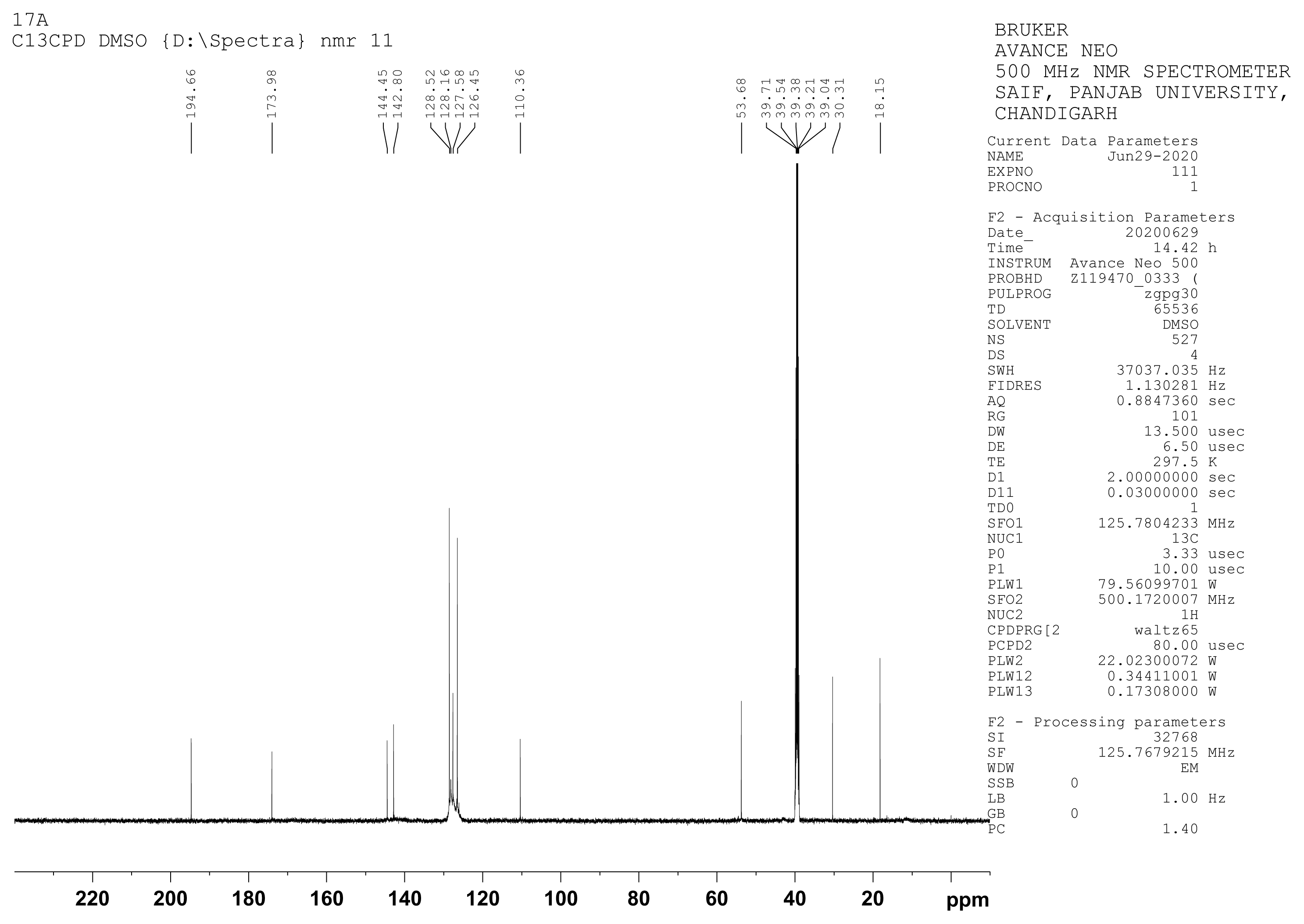

Supplement: Figure 8S — 13C-NMR: 1-(2-hydrazinyl-6-methyl-4-phenyl-1,4-dihydropyrimidin-5-yl) ethenone, (6a) [file turkjchem-45-6-1980s8.tif]

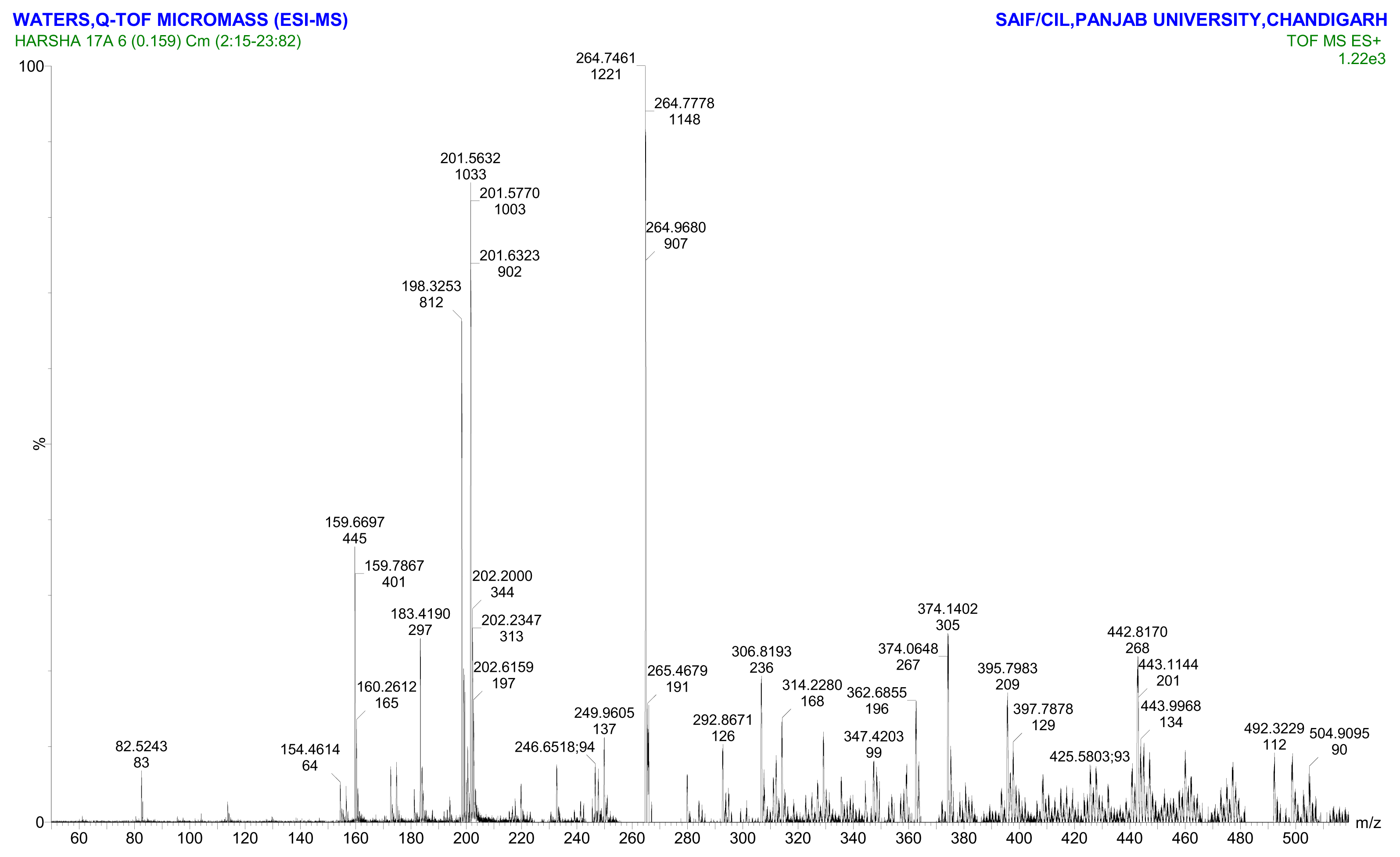

Supplement: Figure 9S — ESI-MS: 1-(2-hydrazinyl-6-methyl-4-phenyl-1,4-dihydropyrimidin-5-yl) ethenone, (6a) [file turkjchem-45-6-1980s9.tif]

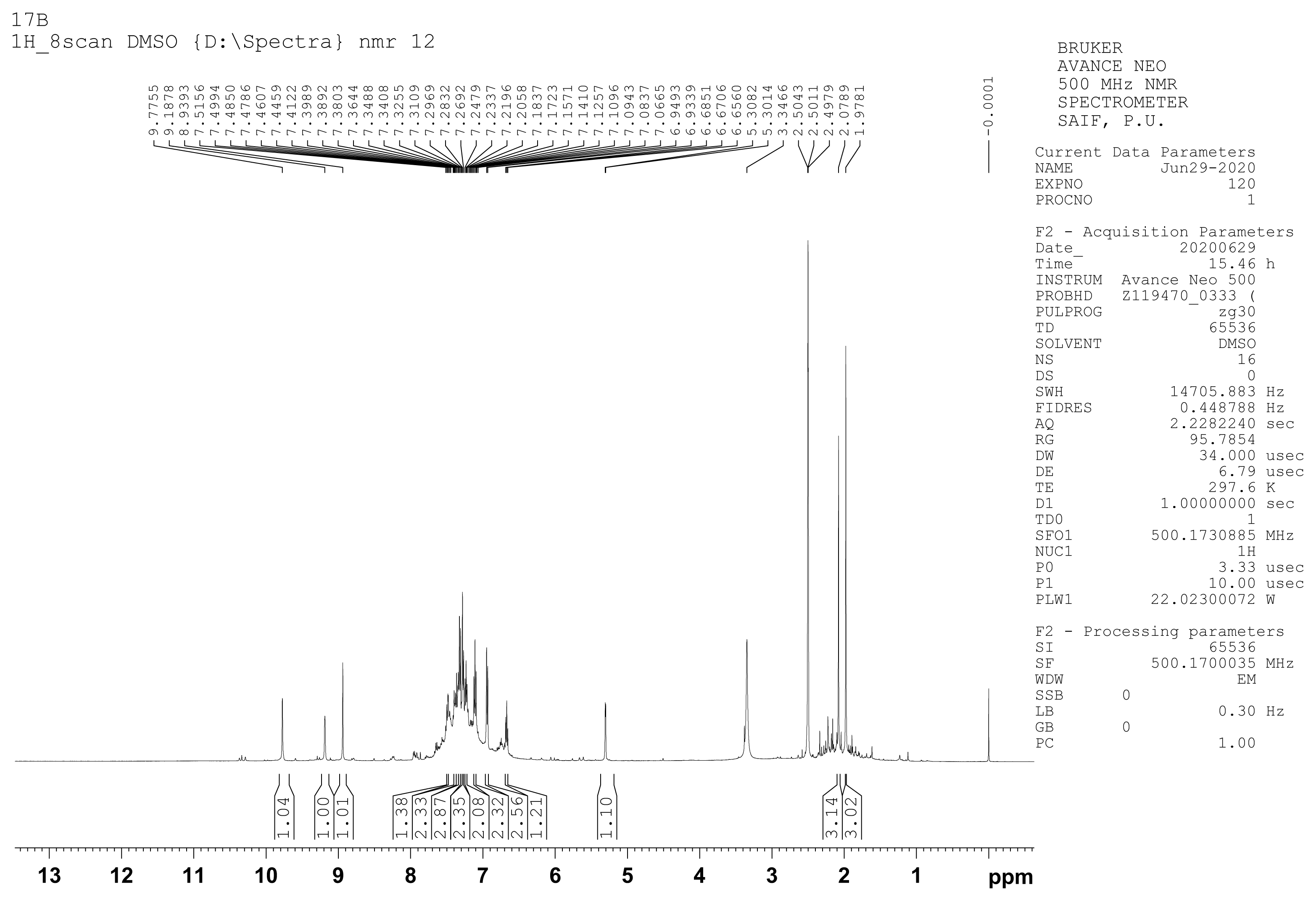

Supplement: Figure 10S — 1H-NMR: 1-[6-methyl-4-phenyl-2-(2-phenylhydrazinyl)-1,4-dihydropyrimidin-5-yl]ethenone (6b) [file turkjchem-45-6-1980s10.tif]

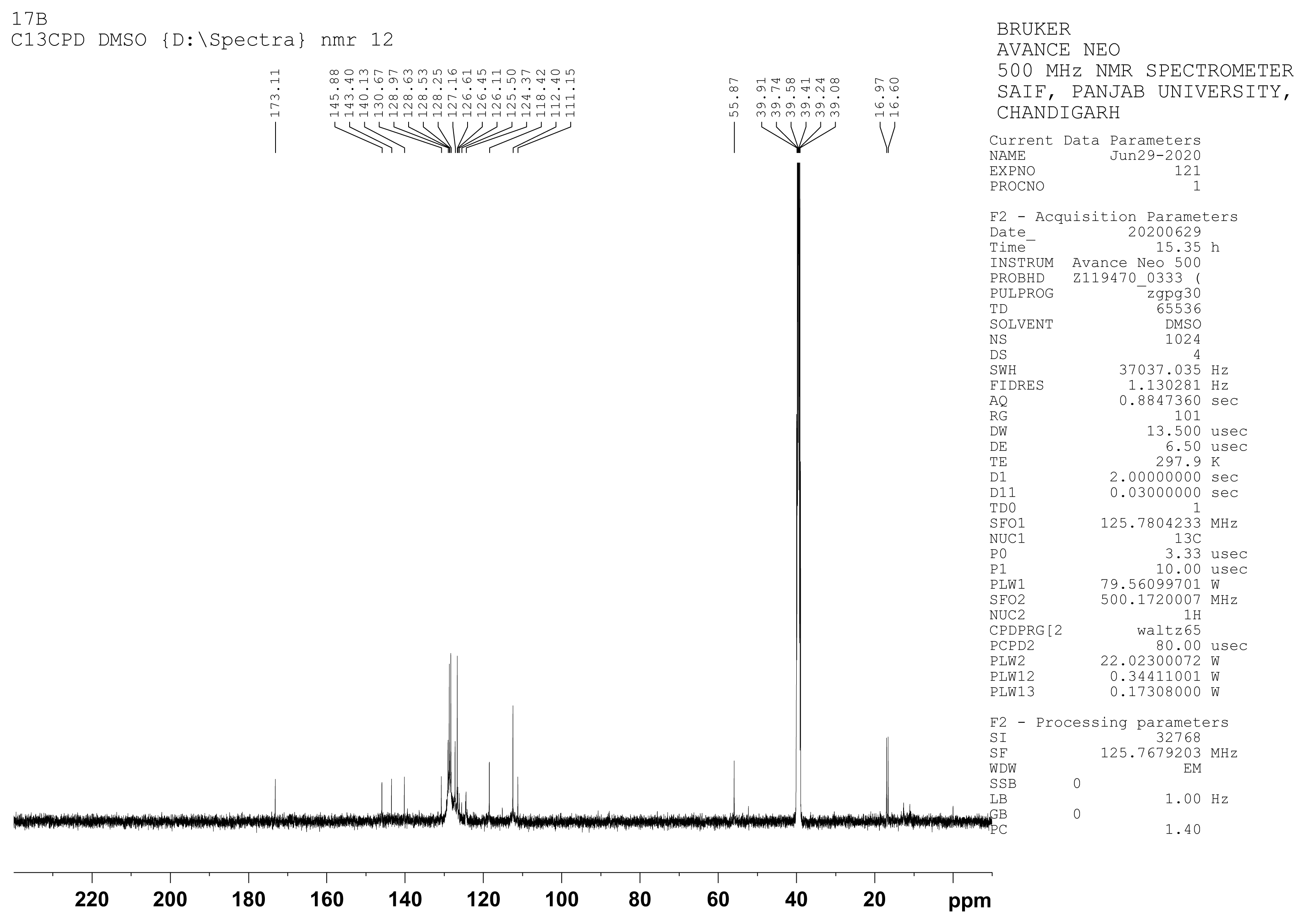

Supplement: Figure 11S — 13C-NMR: 1-[6-methyl-4-phenyl-2-(2-phenylhydrazinyl)-1,4-dihydropyrimidin-5-yl]ethenone (6b) [file turkjchem-45-6-1980s11.tif]

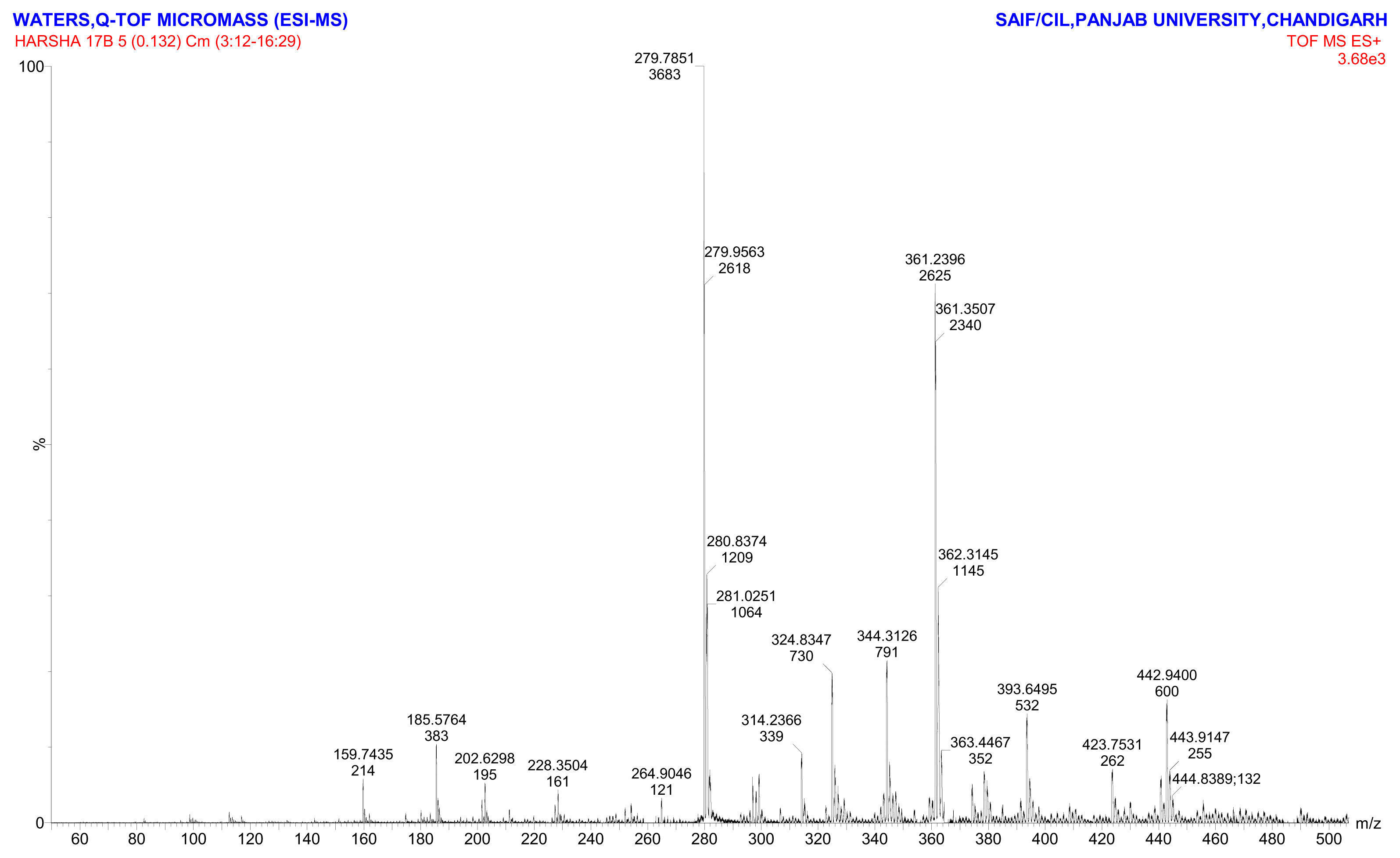

Supplement: Figure 12S — ESI-MS: 1-[6-methyl-4-phenyl-2-(2-phenylhydrazinyl)-1,4-dihydropyrimidin-5-yl]ethenone (6b) [file turkjchem-45-6-1980s12.tif]

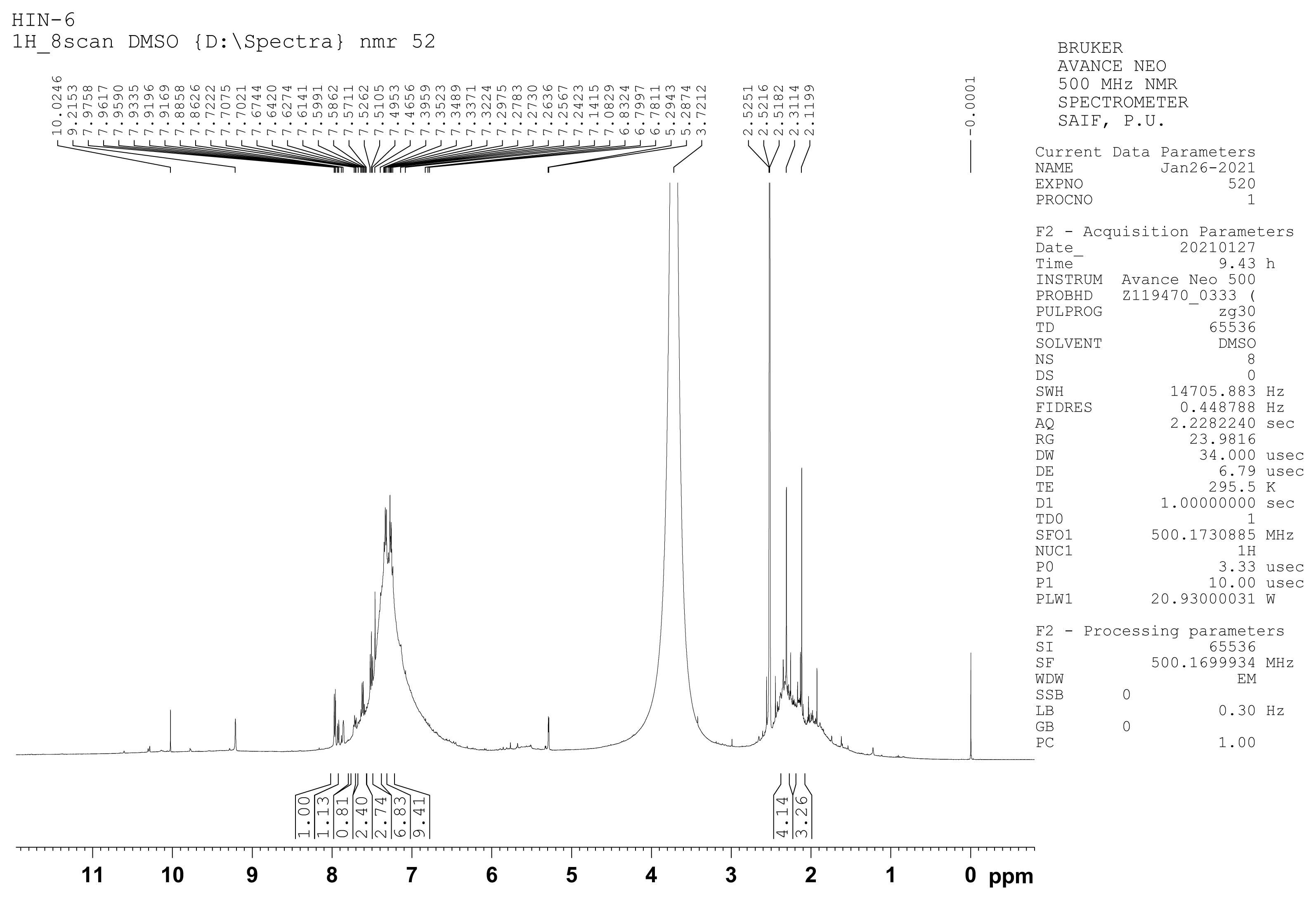

Supplement: Figure 13S — 2-(5-acetyl-6-methyl-4-phenyl-1,4-dihydropyrimidin-2-yl)hydrazinecarboxamide (6c) 1H-NMR: [file turkjchem-45-6-1980s13.tif]

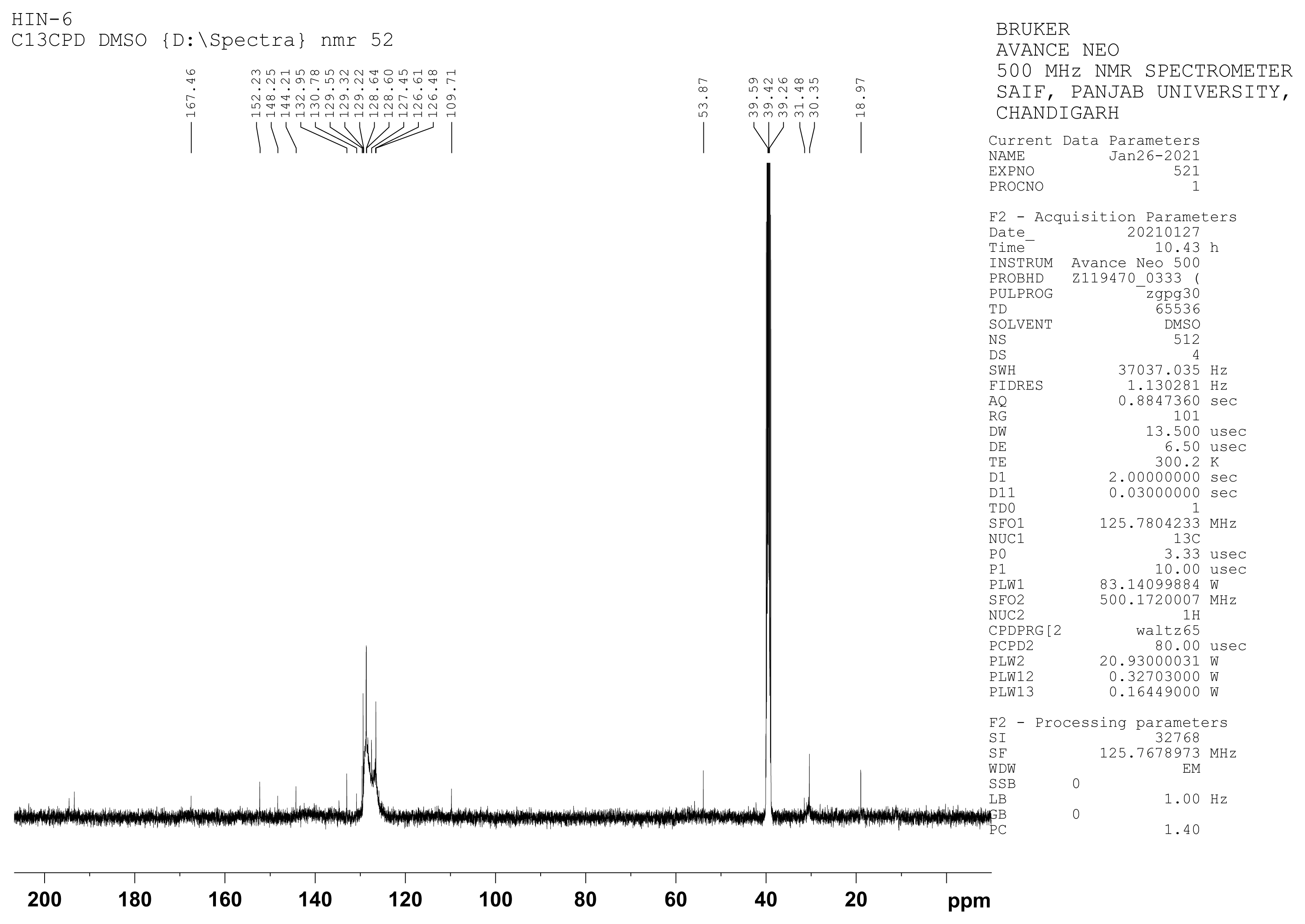

Supplement: Figure 14S — 13C-NMR: 2-(5-acetyl-6-methyl-4-phenyl-1,4-dihydropyrimidin-2-yl)hydrazinecarboxamide (6c) [file turkjchem-45-6-1980s14.tif]

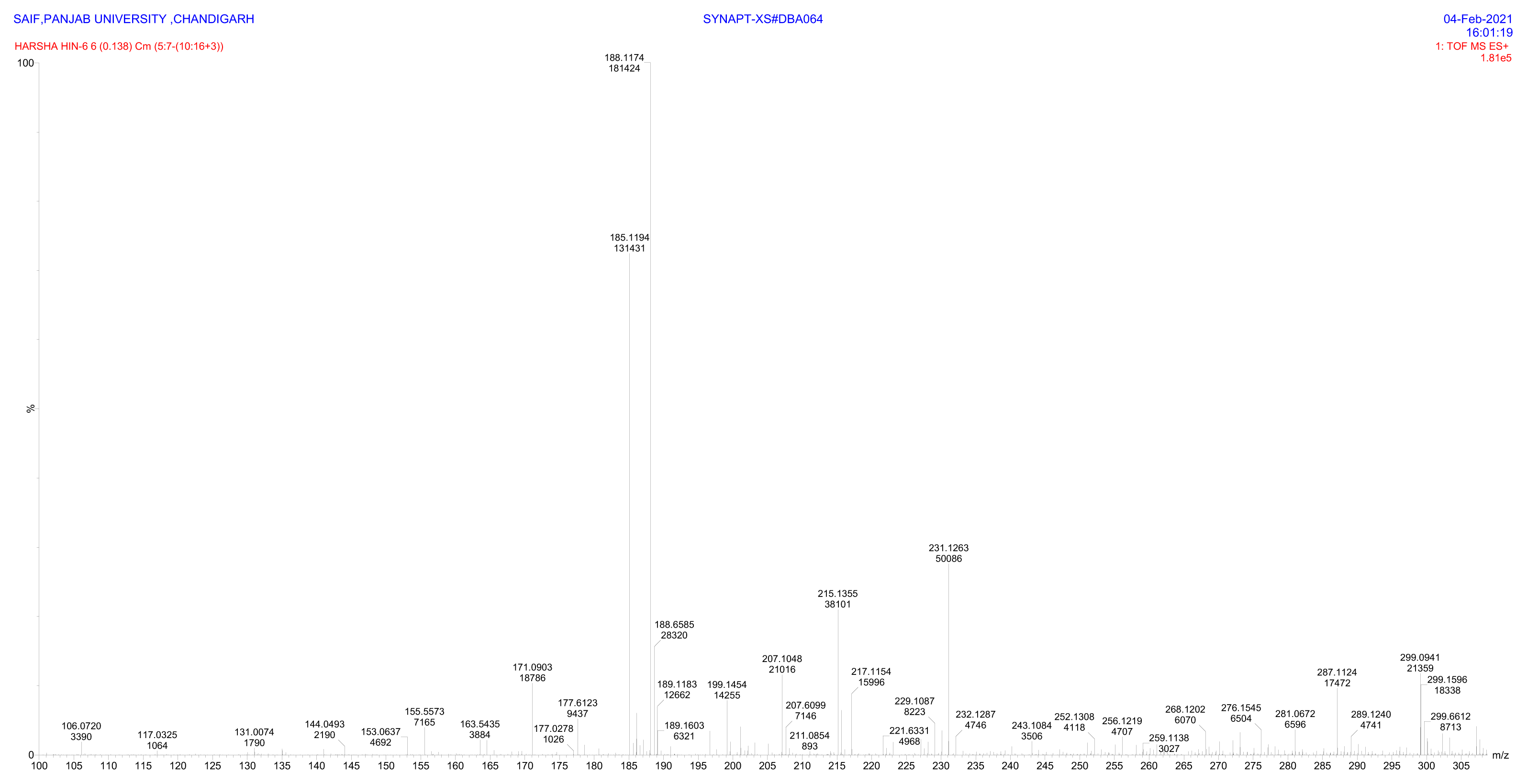

Supplement: Figure 15S — ESI-MS: 2-(5-acetyl-6-methyl-4-phenyl-1,4-dihydropyrimidin-2-yl)hydrazinecarboxamide (6c) [file turkjchem-45-6-1980s15.tif]

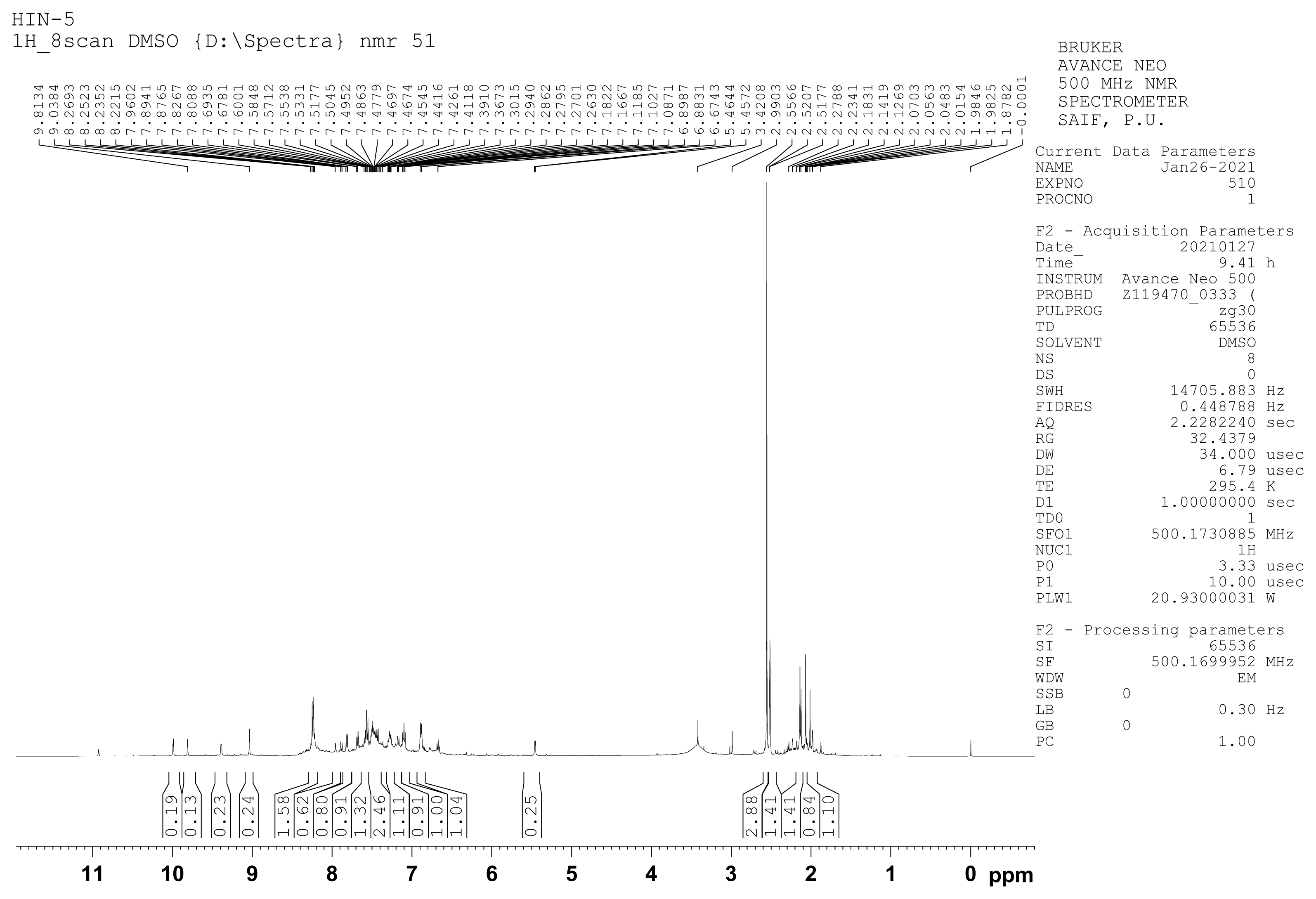

Supplement: Figure 16S — 1H-NMR: 1-[6-Methyl-4-(4-nitro-phenyl)-2-(N′-phenyl-hydrazino)-1,4-dihydro-pyrimidin-5-yl]-ethanone (6d) [file turkjchem-45-6-1980s16.tif]

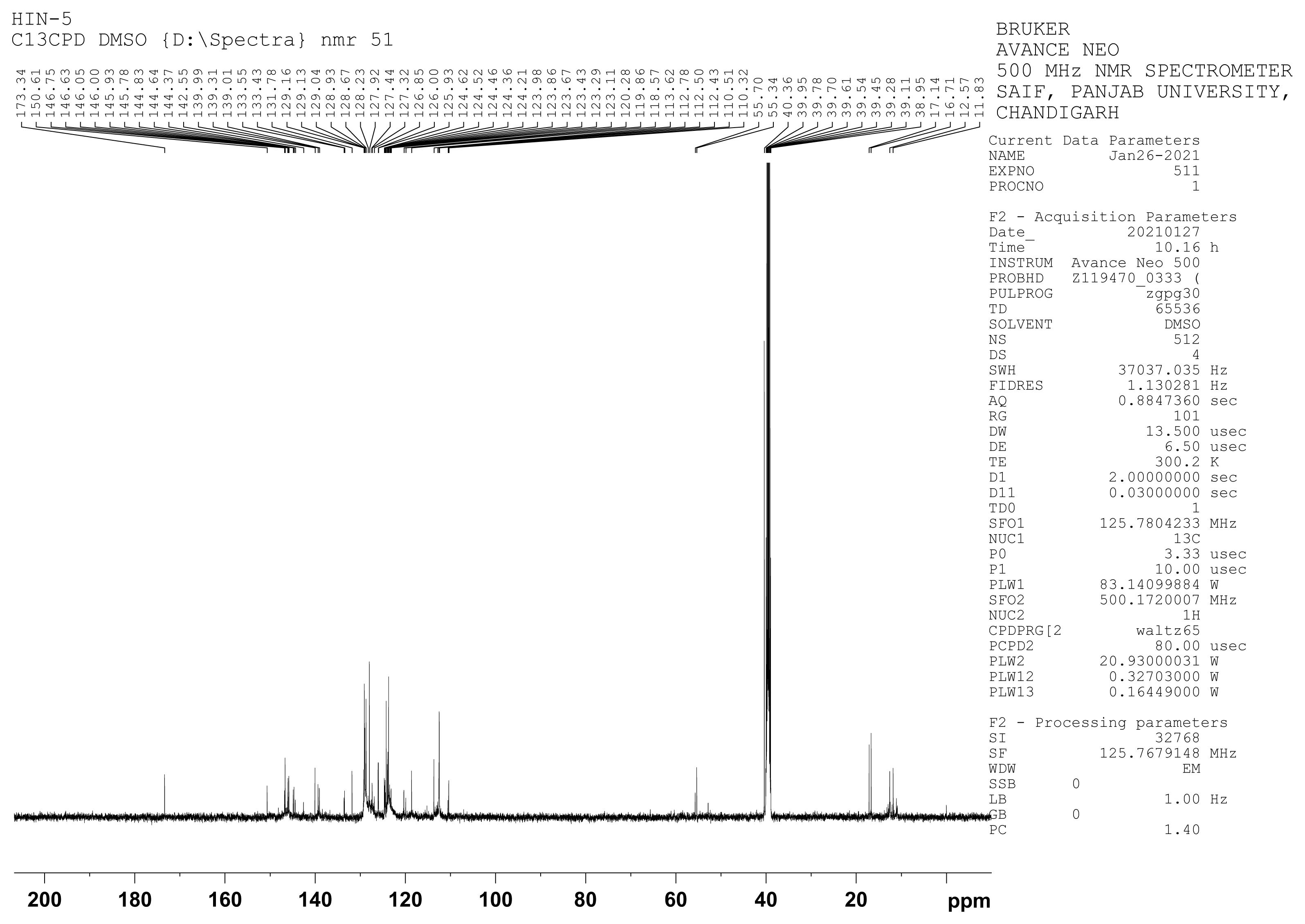

Supplement: Figure 17S — 13C-NMR: (6d) [file turkjchem-45-6-1980s17.tif]

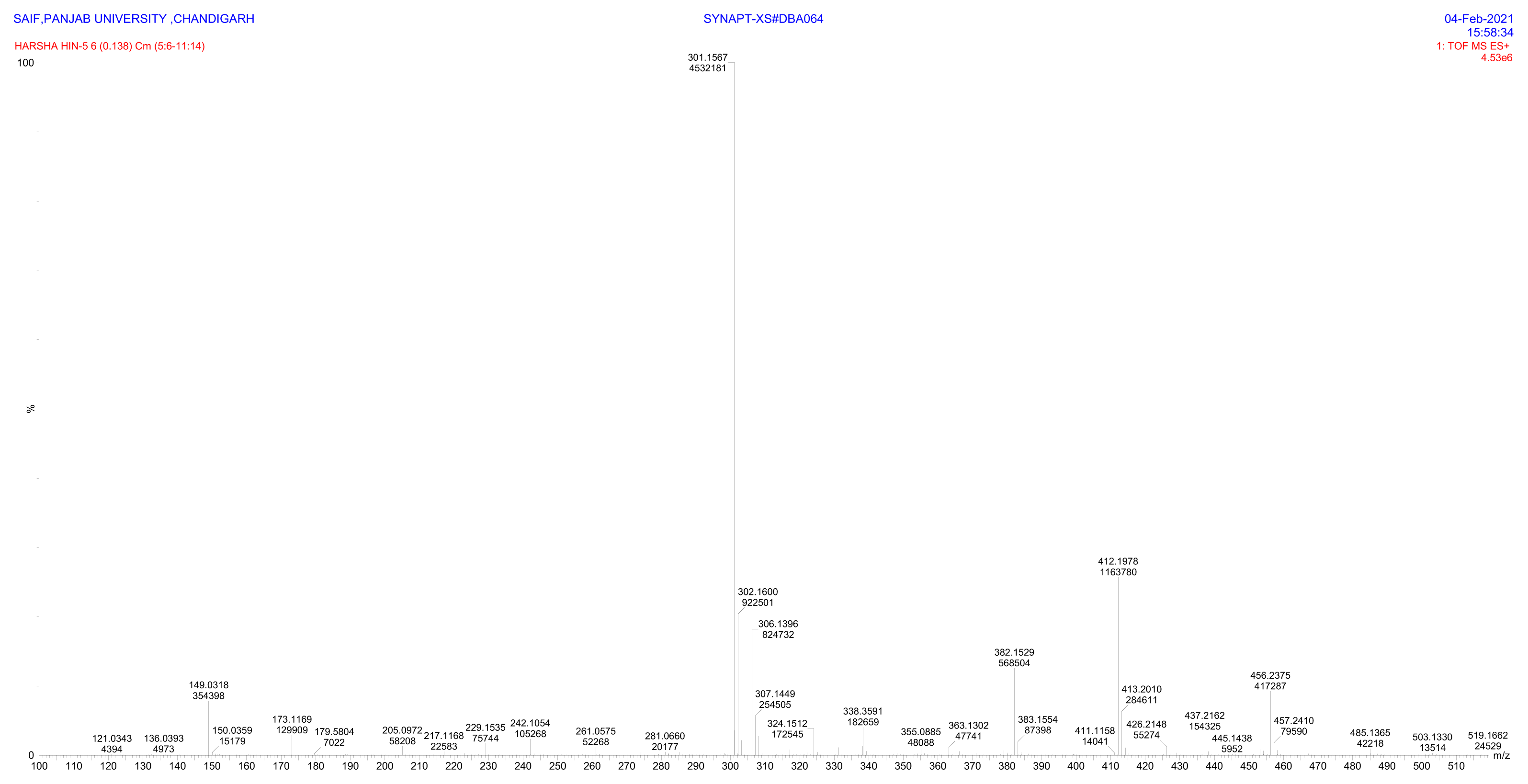

Supplement: Figure 18S — ESI-MS: (6d) [file turkjchem-45-6-1980s18.tif]

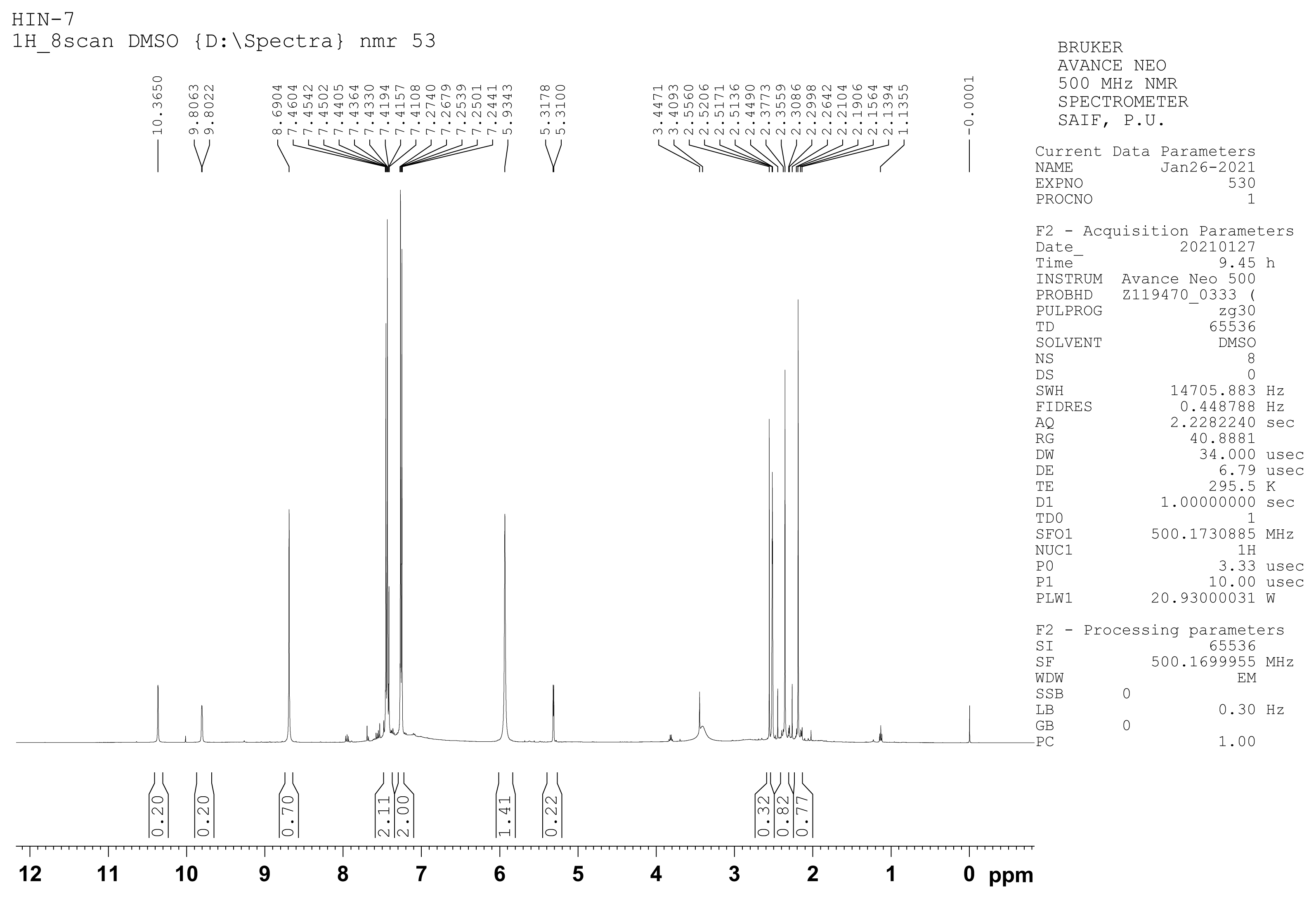

Supplement: Figure 19S — 1H-NMR: 2-[5-acetyl-4-(4-chlorophenyl)-6-methyl-1,4-dihydropyrimidin-2-yl]-N-(4-chlorophenyl)-hydrazinecarboxamide (6e) [file turkjchem-45-6-1980s19.tif]

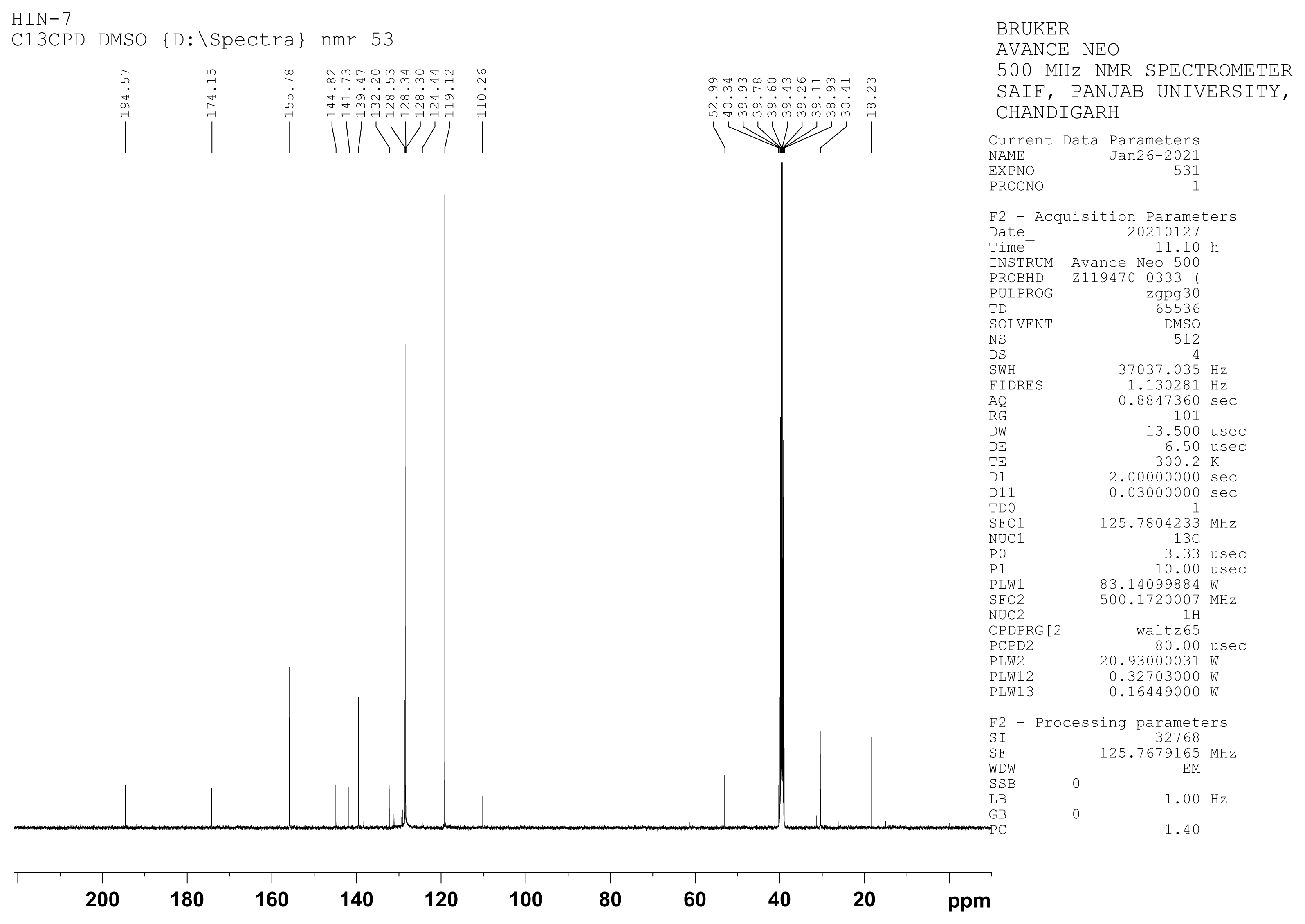

Supplement: Figure 20S — 13C-NMR: (6e) [file turkjchem-45-6-1980s20.tif]

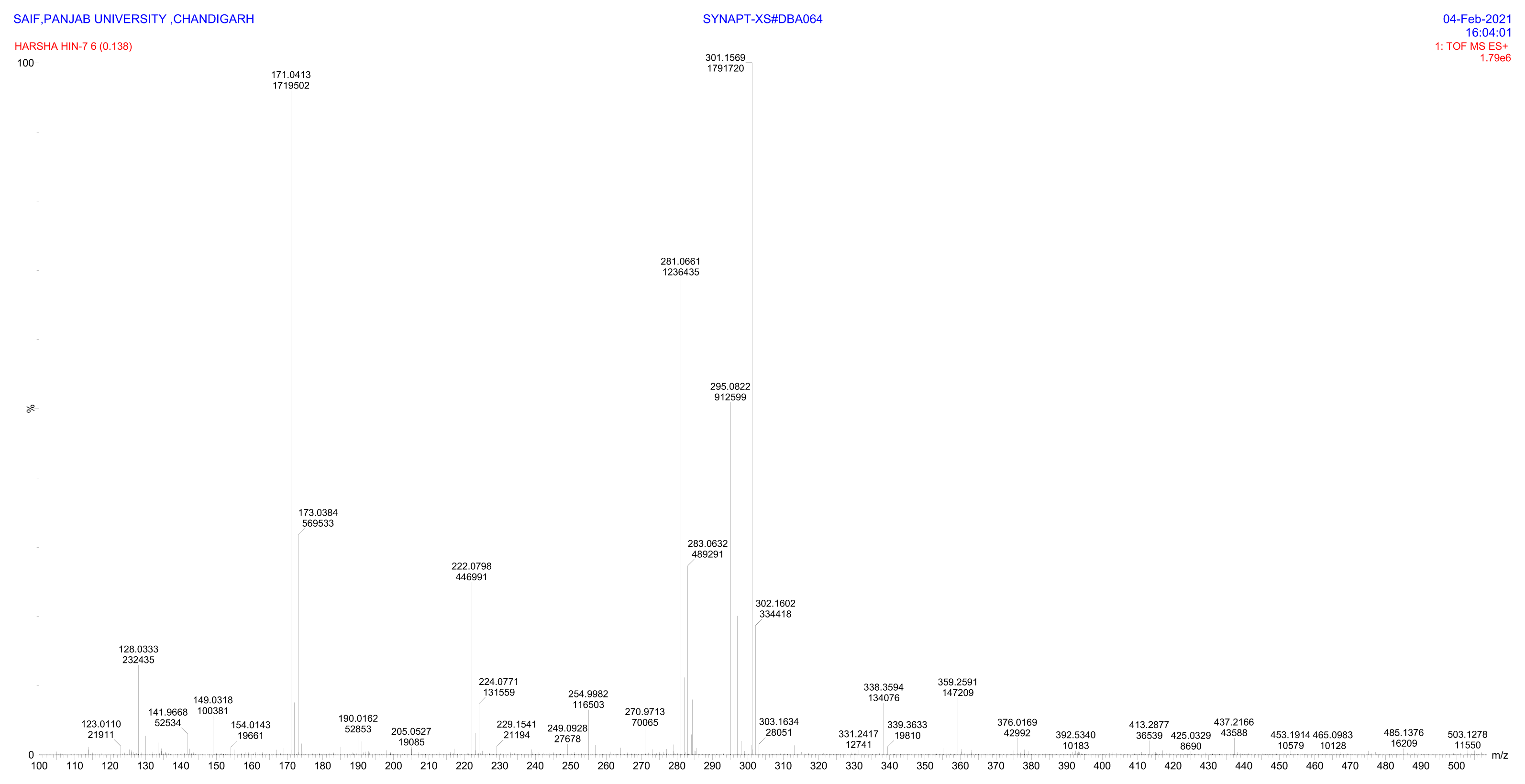

Supplement: Figure 21S — ESI-MS: (6e) [file turkjchem-45-6-1980s21.tif]
